# Compatibility Effects With Simple Lever Tools: A Replication and Extension Beyond Simple Button Responses

**DOI:** 10.1177/00187208261463136

**Published:** 2026-07-02

**Authors:** Valentin Koob, Markus Janczyk

**Affiliations:** 1 9168 University of Bremen, Bremen, Germany

**Keywords:** response–effect compatibility, stimulus–effect compatibility, stimulus–response compatibility, laparoscopic surgery, action effects

## Abstract

**Objective:**

We examined how response–effect (RE), stimulus–response (SR), and stimulus–effect (SE) compatibility jointly influence performance in lever tool use, and test the robustness of previous results across different input modalities.

**Background:**

According to the ideomotor principle, motor actions are selected via anticipating their effects. This becomes particularly relevant in tool use, where the relationship between hand movement and tool movement might be inverted. While various compatibilities are known to influence performance, their interactions remain poorly understood.

**Method:**

We built upon work by Müsseler and Skottke (2011) using orthogonal manipulations of RE, SR, and SE compatibility. Across four experiments with student samples (2022–2024), we varied input modality (button presses, continuous sliders, touchless gestures) and lever rotation type (discrete vs. continuous) to assess the generalizability of the original findings.

**Results:**

Müsseler and Skottke (2011) showed that the interaction of SR and SE compatibility depends on the RE compatibility condition. Consistent with this, we found that under RE compatible conditions, performance was improved when SR and SE compatibilities aligned. However, under RE incompatible conditions, the SR × SE interaction disappeared with button press responses (Experiments 1 and 2) or reversed with more continuous responses (Experiments 3 and 4).

**Conclusion:**

These findings highlight the dynamic interplay of various compatibility relations for untrained participants, and that this interplay depends on, for example, the input device.

**Application:**

Our results inform the design of human-tool interfaces by highlighting when aligning response–effect mappings benefits performance and when mismatches can alter or even reverse other compatibility relationships.

## Introduction

Human motor actions are performed to bring about intended outcomes in the world. Whether flipping a switch to turn on a light or pulling a control lever to guide an aircraft, effective motor control relies on the ability to anticipate how specific movements influence the surrounding environment. Anticipation of a motor action’s consequence(s) is at the core of the *ideomotor principle* (Greenwald, 1970; Harleß, 1861; Hommel et al., 2001; Janczyk et al., 2023; Janczyk & Kunde, 2020; Pfister & Janczyk, 2012; Stock & Stock, 2004). According to this idea, action selection (and execution) is achieved by accessing the mental representation of the anticipated outcome or, more generally, the anticipated *action effect*. These representations may comprise body-related effects, such as the tactile and proprioceptive feedback when grasping and moving a handle, but also more external effects, such as the light turning on after a button press or the visually perceived movement of the tip of a lever. These different types of effects have already been distinguished by James (1890).

Using tools is a particularly interesting ability for several reasons. First, tool use has sometimes been seen as something special to humans (see Johnson-Frey, 2003, and also Dani & Ramsey, 2025, for a recent discussion of definitions of tool use in humans and beyond). Second, tools are of utmost interest for applied research as they are common input devices in the modern world of work. Third, tools dissociate the endpoint of the bodily effector from the location of the effective part of the tool. As an example, consider laparoscopic surgery (see Hewitson et al., 2025, for a recent review), where a surgeon manipulates an endoscopic instrument through a small incision in the abdomen. This represents a lever with one pivot point and (1) the hand and the tip of the tool are spatially decoupled and, even worse, (2) moving the hand into one direction results in the instrument’s tip moving into the opposite direction. This is also referred to as the “fulcrum effect” (Gallagher et al., 1998), an example of a tool-based sensorimotor perturbation in the classification of Hewitson et al. (2025).

The present study extends previous work on tool use with the example of a one-pivot lever and focuses on how the relationships between stimulus, hand movement, and action effects shape the performance when using a lever (see e.g., Janczyk, Pfister, & Kunde, 2012; Kunde et al., 2007, 2012; Müsseler et al., 2008). Based on work from human action control and human factors, the three mentioned components and their compatibility relations are critical for lever performance. However, in most studies, only (one or) two of them were manipulated and the third one results from their combinations. An exception to this is the study by Müsseler and Skottke (2011), which served as the starting point for the series of experiments we report in this manuscript. That study will be introduced in more detail below, after we introduce the compatibility relations between stimuli, responses, and effects. The final part of the introduction provides an overview on the present study and its purpose.

### Compatibility Between Stimuli, Responses, and Effects

A wealth of studies exist on compatibility phenomena between stimuli (S) and responses (R), showing that some SR combinations align well with each other and facilitate responding, while others do not and slow responses down. In the context of tools, the effects (E) ensuing from R are important as well, as R and E are often decoupled.

In this regard, a first relationship of interest was just outlined in the example of laparoscopic surgery: the relationship between hand movement and the effect points of a lever matters. In basic research, this relation is indeed often referred to as *response–effect (RE) compatibility* (see Kunde, 2001). An application to handling a lever was reported in two experiments of Kunde et al. (2007), in which participants controlled a virtually presented lever that was fixed midpoint to create a pivot. The task was to move the tip of the lever either toward or away from a stimulus. This mirrors the challenges faced in laparoscopic surgery, where a surgeon must precisely maneuver an instrument toward a target for treatment or away to prevent harm. In one condition, participants controlled the lower end of the lever so that hand and lever tip moved in opposite directions, the *RE incompatible condition* (see Fig. 1, left panel). In a second condition, participants directly controlled the tip of the lever so that hand and lever tip movement were identical, the *RE compatible condition* (see Fig. 1, right panel). Responses were slower and less accurate when the relationship between hand movement and the movement of the effective part of the tool was incompatible relative to when it was compatible. This observation of an RE compatibility effect is expected against the background of the ideomotor principle and has been observed in other studies on lever usage (Janczyk, Pfister, & Kunde, 2012; Kloss & Kunde, 2026; Kunde et al., 2012; Müsseler et al., 2008; Müsseler & Skottke, 2011; Wirth et al., 2016). However, RE compatibility is a rather broad and general phenomenon and has as well been observed in a variety of studies with different stimuli and responses, for example, with simple response key presses (e.g., Janczyk & Lerche, 2019; Kunde, 2001; Paelecke & Kunde, 2007; Pfister & Kunde, 2013), sequences of key presses and effects (e.g., Brown et al., 2022; Brown & Koch, 2024; Janczyk et al., 2017; Keller & Koch, 2008), wheel rotations and rotating effects (Janczyk et al., 2015; Janczyk, Pfister, Crognale, & Kunde, 2012; Yamaguchi & Proctor, 2011), or vocal responses (e.g., Földes et al., 2018; Koch & Kunde, 2002). Despite this generality, our focus here is on lever usage and the emerging compatibility effects.

**Figure 1.**
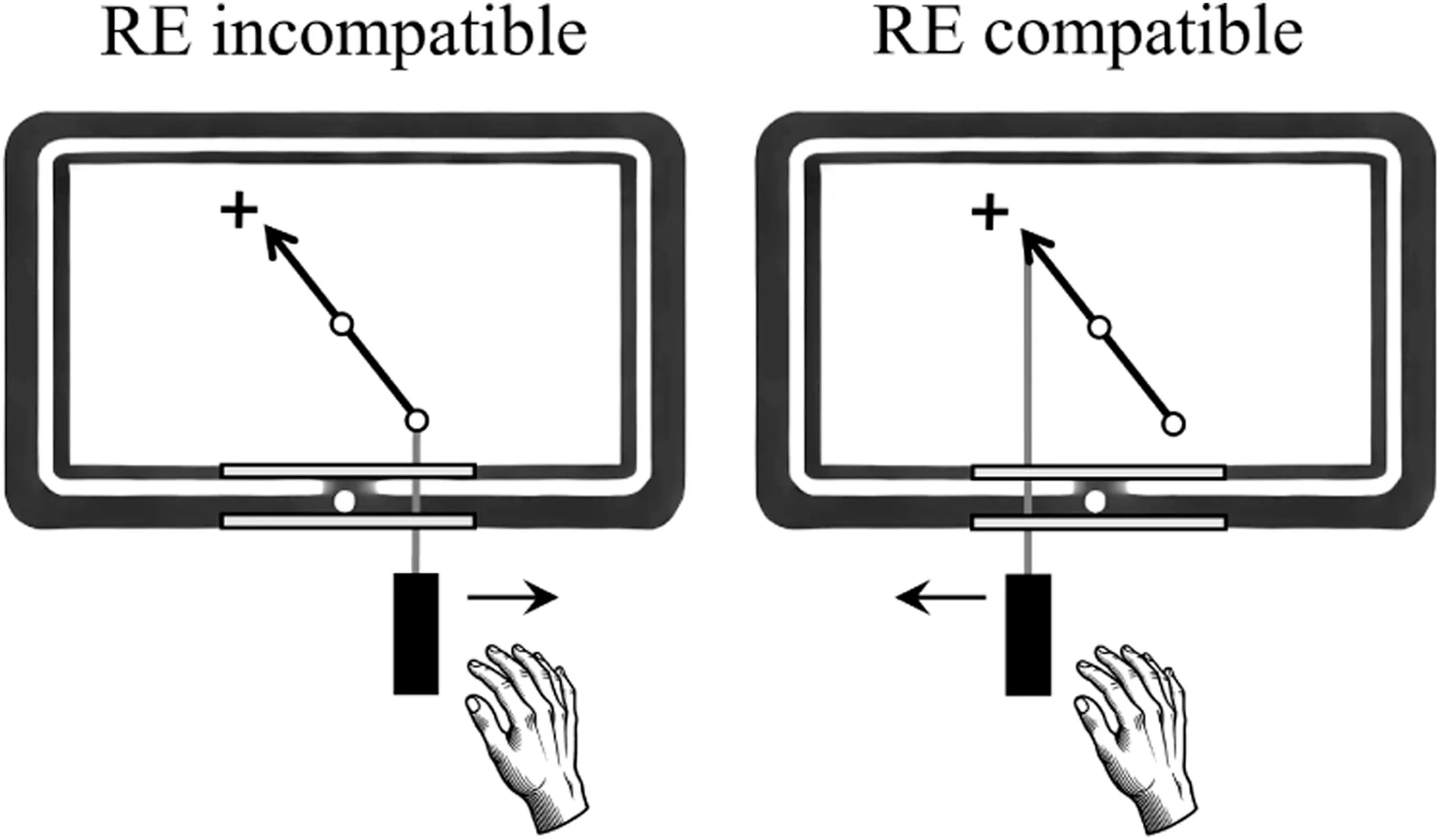
Simplified illustration of the experimental setup used by Kunde et al. (2007). *Note.* Participants controlled a lever fixed at its midpoint with one pivot. The lever was presented virtually and manipulated using a physical slider positioned directly in front of a screen. When controlling the handle of the lever (left panel), moving the hand to the right caused the lever’s tip to move to the left. In contrast, when controlling the tip of the lever (right panel), both the hand and the lever tip moved into the same direction. Hence, the left panel illustrates an RE incompatible condition and the right panel illustrates an RE compatible condition. In addition, the left panel visualizes an SR incompatible and SE compatible condition, while the right panel illustrates an SR compatible, and SE compatible condition. RE = response–effect, SR = stimulus–response, SE = stimulus–effect

Second, it is well established that performance is improved when stimulus and response location match compared with when they mismatch. This phenomenon is known as *stimulus–response (SR) compatibility* (Fitts & Deininger, 1954; Fitts & Seeger, 1953; for a review, see Proctor & Vu, 2006). In an *SR compatible* trial, the response to a left stimulus would also be left, while in an *SR incompatible* trial, participants would need to give a right response to the left stimulus. If SR compatible and incompatible conditions are applied block-wise, SR compatible responses are faster and less error prone than are SR incompatible responses. While in SR compatibility studies, location is the task-relevant feature that determines the correct response, a similar effect persists even when stimulus location is irrelevant. If, for example, responses are determined by the color of a stimulus that appears in a left or right location (which is now task-irrelevant), responses are still faster and less-error prone in SR compatible conditions where stimulus and response location match. This result is known as the Simon effect (Simon, 1969). The common explanation is that a stimulus can automatically activate a response on the same side, thereby enhancing performance in corresponding conditions, but creating interference that must be resolved in incompatible conditions (see Janczyk et al., 2026; Ulrich et al., 2015, for a modeling perspective on this).

Finally, performance can also be affected by the compatibility between the effective part of the lever and the stimulus, hence by *stimulus–effect (SE) compatibility*. When the lever’s effect point must be moved toward the target stimulus, this is an *SE compatible* relationship. Conversely, if the lever’s effect point must be moved away from the target stimulus, the relationship is *SE incompatible*. How the SE compatibility influences performance when using a lever will be summarized further below.

A key challenge in investigating how RE, SR, and SE compatibility influence performance is that these compatibility types cannot be manipulated independently when using a simple lever. To illustrate this, consider again the setup by Kunde et al. (2007), which involved a simple one-pivot lever as is illustrated in Figure 1. A plus-sign as the stimulus indicates that the participant is required to move the tip of the lever toward the stimulus. When participants grasp the lever at the handle (left panel), the RE relation is incompatible because the tip of the lever and the hand move in opposite directions. The SR relation in that panel is also incompatible because the hand must move to the right to reach the target on the left side. However, this necessarily implies a compatible SE relationship because the effective part of the tool moves toward the stimulus. When instead participants directly control the tip of the lever (right panel), the RE relationship is compatible. However, this change does not only affect the RE relationship. If a participant now aims to move the tip of the lever to the left toward stimulus, the hand must move left as well and hence this is an SR compatible condition. Thus, the SR relation is compatible, while the SE relation remains compatible, too. Consequently, when using a simple lever with one pivot, RE, SR, and SE compatibility cannot be manipulated independently and if two relations are known, the third relation is always determined.

### The Study by Müsseler and Skottke (2011)

To address this issue, Müsseler and Skottke (2011) designed an experimental setup that involved two types of levers: a U-shaped and an inverted-U-shaped lever (see Figure 2 for an illustration). By using these levers and instructing participants to move an effective part of the lever either toward or away from the target, this setup allowed orthogonal manipulation of RE, SR, and SE compatibility.

**Figure 2.**
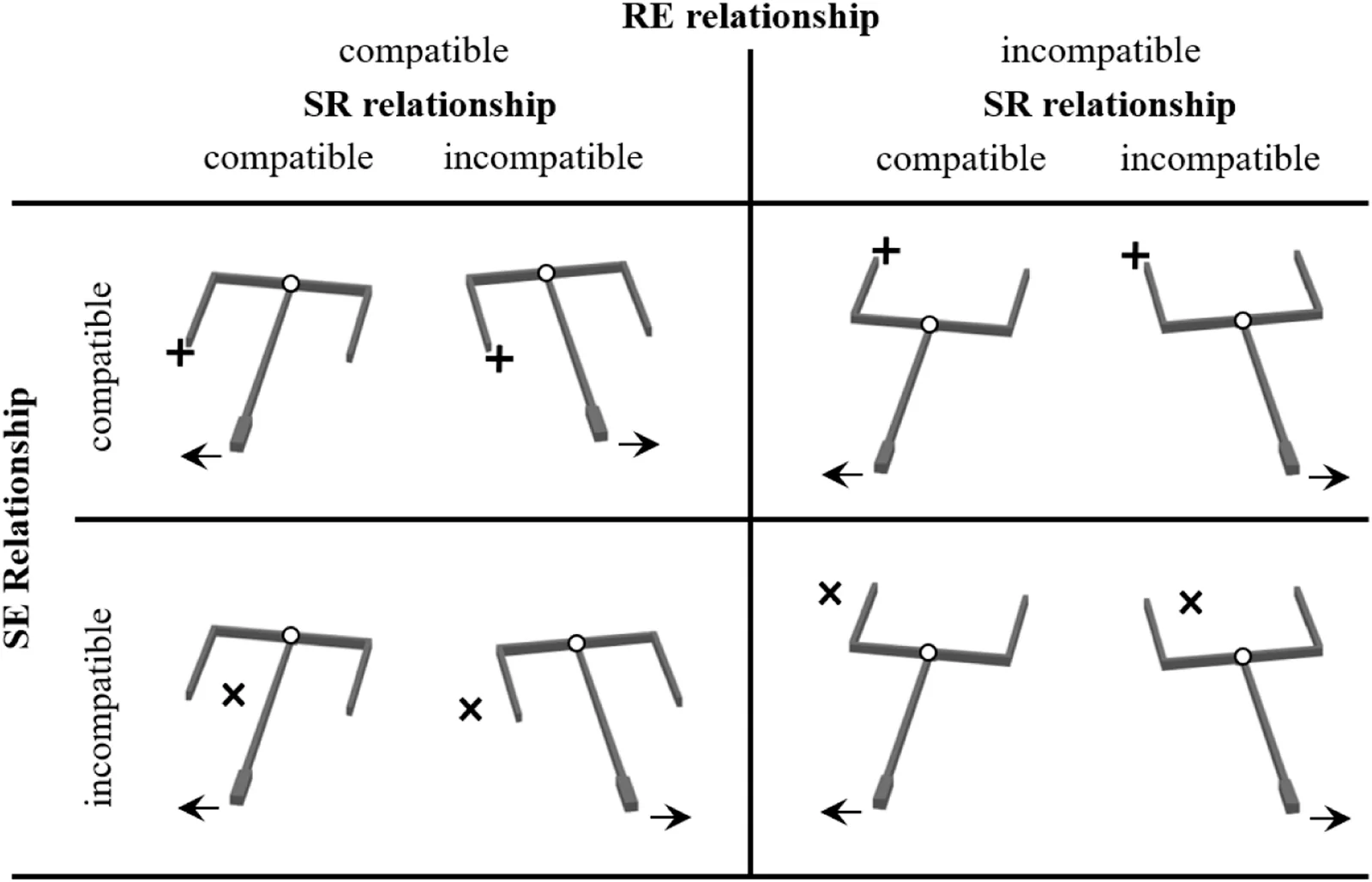
Levers and task as used by Müsseler and Skottke (2011). *Note.* Each combination of target and lever can be classified as RE compatible/incompatible, SE compatible/incompatible, and SR compatible/incompatible, resulting from the relationship between response direction, stimulus position, and the movement of the lever tip. In the study by Müsseler and Skottke (2011), participants had to move the lever’s tip toward (“+” stimulus) or away from the target (“ × ” stimulus; see also Fig. 4 below for the actual stimuli). Arrows indicate the movement of the handle and thus the rotation of the lever (but were not visible in the actual experiment)

Each of the two lever variants consists of a vertical rod with a grip at its lower section and a centrally positioned crossbar at the top. The pivot point is located at the center of the horizontal rod, while the effect points are positioned at the ends of additional rods extending either upward or downward from the crossbar. The primary distinction between the two levers lies in their RE compatibility relationship: the U-shaped lever implements an incompatible RE relationship, as hand movements are transformed into opposite tool-effect movements. In contrast, the inverted-U-shaped lever implements a compatible RE relationship, as hand and tool-effect movements go into the same direction.

The results obtained by Müsseler and Skottke (2011) for response time (RT) and percent error (PE) are re-presented in Figure 3, as they are of particular importance to the present study. With the inverted-U-shaped lever, and hence a compatible RE relationship, SR compatibility facilitated performance when the SE relationship was compatible as well. When, in contrast, the SE relationship was incompatible, SR compatibility had a reversed effect. Thus, performance was best, whenever SR and SE compatibility matched. When using the U-shaped lever and an incompatible RE relationship, overall performance declined, thus being an RE compatibility effect. More importantly, however, the disordinal interaction between SR and SE compatibility disappeared and performance did not much depend on SR and SE compatibility. The authors argued that participants, when using tools with a compatible RE relationship, focus mainly on the tool’s effects rather than on their own hand movements. In this case, they barely notice the transformation between hand and tool movements, allowing automatic processes to dominate and enabling SR and SE relationships to exert an influence. However, when the transformation is inverse and thus incompatible, users must invest much more attention to controlling hand movements, causing slower intentional processes to override the automatic processes of motor control (see p. 389).

**Figure 3.**
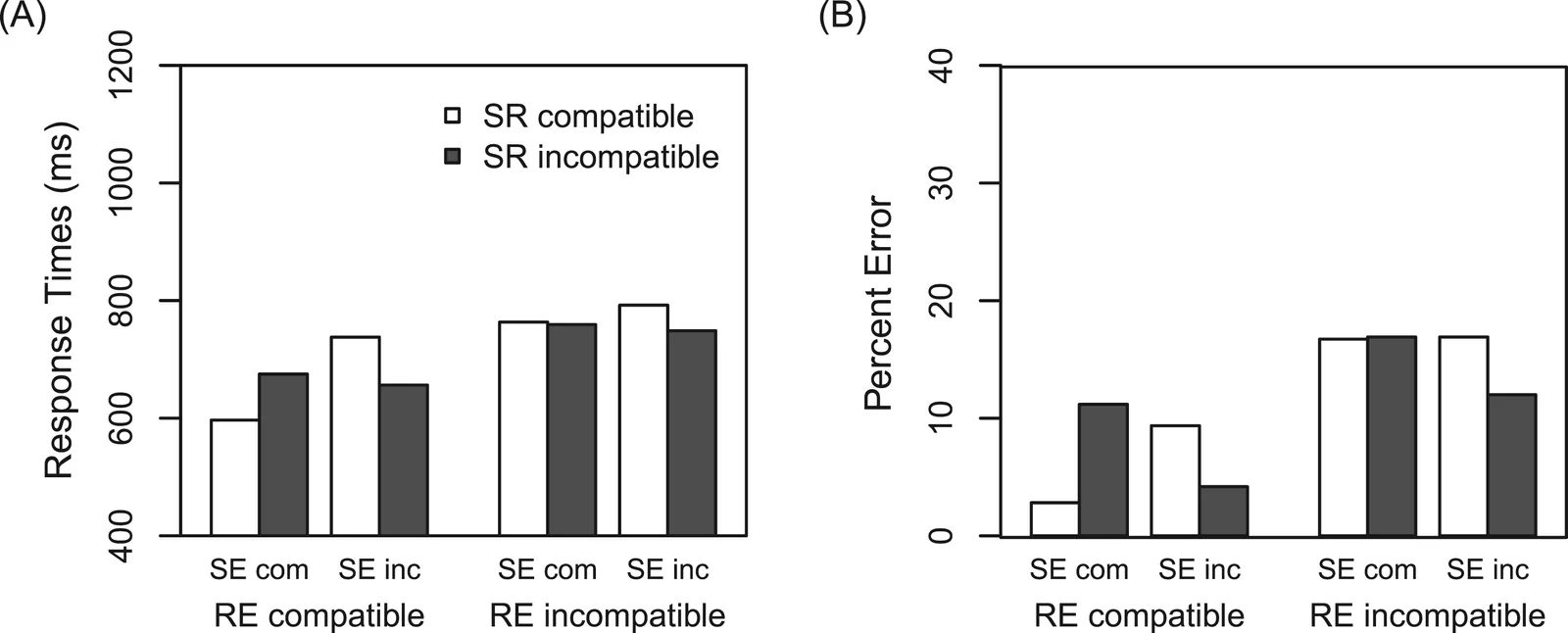
Re-presentation of the results by Müsseler and Skottke (2011). *Note.* Panels (A) and (B) show the results for RTs and PEs as a function of RE, SR, and SE compatibility. White and dark gray bars represent compatible and incompatible SR relationships, respectively. The values in this graph were obtained from Figure 5 of Müsseler and Skottke (2011)

### Purpose and Overview of the Present Study

The present study aims to replicate and extend the study of Müsseler and Skottke (2011) in several ways in order to examine the stability of the reported three-way interaction between RE, SR, and SE compatibility. More precisely, the present study was motivated in three ways and presents four experiments. These experiments are summarized in Table 1, where we also summarize the core findings of all upcoming experiments.

**Table 1.** General Summary of Experiments and Their Results

Experiment	Input	Rotation	Interactions		
			3-way	2-way (RE comp)	2-way (RE incomp)
1	Keys	Abrupt	Yes	Yes	No
2	Keys	Continuous	Yes	Yes	No/yes
3	Slider	Continuous	Yes	Yes	Yes
4	Gestures	Abrupt	Yes	Yes	Yes

*Note.* This table summarizes how participants controlled the levers (column Input) and the continuity of the lever rotation (column Rotation). The last three columns provide a summary of the core results of each study with respect to the presence or absence of a three-way interaction among SE, SR, and RE compatibility, as well as the two-way interactions between SE and SR compatibility at each level of RE compatibility. The entry no/yes for Experiment 2 highlights that the 2-way interaction for RE incompatible relationships was only present for some key dependent variables but not for RTs.

First, upon reading the original results, one might indeed be surprised that the SR and SE compatibility effects were overruled by the incompatible RE relationship, as both SR and SE effects are remarkably robust in other studies, even under RE incompatible conditions (e.g., Janczyk, Pfister, & Kunde, 2012; Kunde et al., 2007, 2012). One explanation for this result might be pure chance. In fact, Müsseler and Skottke (2011) only ran a single experiment with *n* = 10 participants, and we are unaware of any other study that has utilized the (inverted)-U-lever subsequently. The absent SE × SR interaction with an RE incompatible lever might thus be a false negative result. As a consequence, we aimed to replicate the original study with a larger sample in an attempt to further corroborate the original result of Müsseler and Skottke (2011) in Experiment 1.

Second, a potential objection to the original setup is that participants controlled the levers with simple button presses. Specifically, on each trial, the lever started in a central position with its handle perfectly aligned vertically. After the imperative stimulus occurred, pressing a left or right button immediately rotated the lever to the corresponding end position. As a result, lever movements were abrupt, and the input responses were discrete rather than continuous. This may have created a highly salient relationship between responses and effects, which could have been strong enough to override the SR and SE compatibility effects. To address this potential drawback, we also used continuous lever rotations in Experiment 2. Apart from being a first step into the direction of continuous responses and effects, another purpose of this experiment is to (again) provide an independent conceptual replication of Müsseler and Skottke (2011) and our Experiment 1.

Third, building on these two first experiments, we examined the stability of the three-way interaction when the set-level compatibility is systematically varied by changing the input device. In a previous study, Janczyk et al. (2015) provided evidence that the RE compatibility effect is influenced by the set-level compatibility between the input device and the response effect. Specifically, participants rotated either an RE compatible or RE incompatible version of an aircraft’s attitude indicator. In one condition, participants controlled the aircraft with left and right joystick movements (low set-level compatibility), while in a second condition, a steering wheel was used instead (high set-level compatibility). The RE compatibility effect was larger with the high than with the low set-level compatibility. Against this background, we tried to increase the RE compatibility effect in Experiment 3 by allowing participants to control the lever continuously with a physical slider placed in front of the monitor. The critical question is whether a potentially stronger RE compatibility effect alters the three-way interaction in any way. This was complemented by Experiment 4, where we examined the influence of a potentially reduced RE compatibility effect. To this end, we used touchless gestures, that is, (left and right) movements of the hand that did not require participants to press a response key or grab a lever slider for responding, as the means to manipulate the lever movement. Previous research has not observed RE compatibility effects with touchless gestures, although SR compatibility and Simon effects were clearly observed with them (Janczyk, 2023; Janczyk et al., 2019). Although the reasons for this are not clear at present, the absence of an RE compatibility effect makes this input method particularly interesting for the present study as well.

## Experiment 1

The goal of Experiment 1 was to replicate the study by Müsseler and Skottke (2011). Any deviations from the original study were limited to rather technical aspects, such as monitor size, refresh rate, viewing distance, or the exact sizing of the lever and target stimuli on the screen.

### Method

#### Transparency and Openness

We report how we determined our sample size, all data exclusions, all manipulations, and all measures in the study, and we follow JARS (Appelbaum et al., 2018). All data, core analyses scripts, experimental source code, and stimulus materials are available on OSF (Koob & Janczyk, 2025; https://osf.io/r93as). The data were analyzed using R (R Core Team, 2022, v. 4.2.2), with the packages tidyverse (Wickham et al., 2019, v. 1.3.2), schoRsch (Pfister & Janczyk, 2016, v. 1.10), and ez (Lawrence, 2016, v. 4.4-0). The design and analyses of this study were pre-registered (see https://aspredicted.org/gcbt-ytxp.pdf). All procedures were in accordance with the Declaration of Helsinki.

#### Participants

We obtained data from students at the University of Bremen until *n* = 40 valid data sets were collected. During sampling in 2022, 11 participants who produced more than 20% erroneous trials in the analyzed test blocks were replaced. Note that we also assessed whether a more liberal cutoff value of 30% would change the present results. In this case, only 8 participants would be excluded, but results did not change. The final sample comprised 32 female and 8 male students, aged 19 to 35 years (*M*_*age*_ = 23.75). From this final sample, one additional participant was excluded due to a high number of error trials in one particular condition (67%) and particularly slow responses compared to other participants. This procedure was not pre-registered, but was applied after visually inspecting the individual data. Including or excluding this participant’s data did not change our conclusions, and we report the results without this participant. The results with the full sample can be found in the OSF repository. All participants received course credit or monetary compensation, provided written informed consent, and had normal or corrected-to-normal vision.

The sample was nearly four times larger than the original sample of *n*_*orig*_ = 10 in Müsseler and Skottke (2011). According to a power analysis, *n* = 39 participants are sufficient to detect an effect size of $\eta_{p}^{2}\geq .22$ with a power of about 1 − *β* = 0.9 (Langenberg et al., 2023). The reported effect size for the three-way interaction for response times (RTs) was $\eta_{p}^{2}=.42$ in the original study. With *n* = 39 participants, we also exceed the (sometimes) recommended sample size for replication studies of *n* = 2.5 ⋅ *n*_*orig*_ (Simonsohn, 2015).

#### Apparatus and Stimuli

Participants were tested individually in a sound-attenuated experimental cabin at the Department of Psychology of the University of Bremen. Stimuli and instructions were presented on a 17-inch monitor (1,024 × 768 pixels), controlled by custom C++ software, run on a standard PC. Stimuli were the original U-shaped and inverted-U-shaped levers shown as pictures. The imperative stimuli, represented as either a “+” or “ × ” symbol, were displayed in white within black ellipses and positioned at either the left or right effect points of the lever (see Figure 4). Two custom-built response buttons, placed to the left and right of the participant, served as response keys. Participants pressed the keys with the index fingers of the respective hand. In contrast to the original study, participants did not place their head on a chin rest. This was done as we aimed to extend the original study with continuous motor movements in subsequent experiments (see Exp. 3 and Exp. 4), where such a chin rest would impair the participants’ freedom of movement.

**Figure 4.**
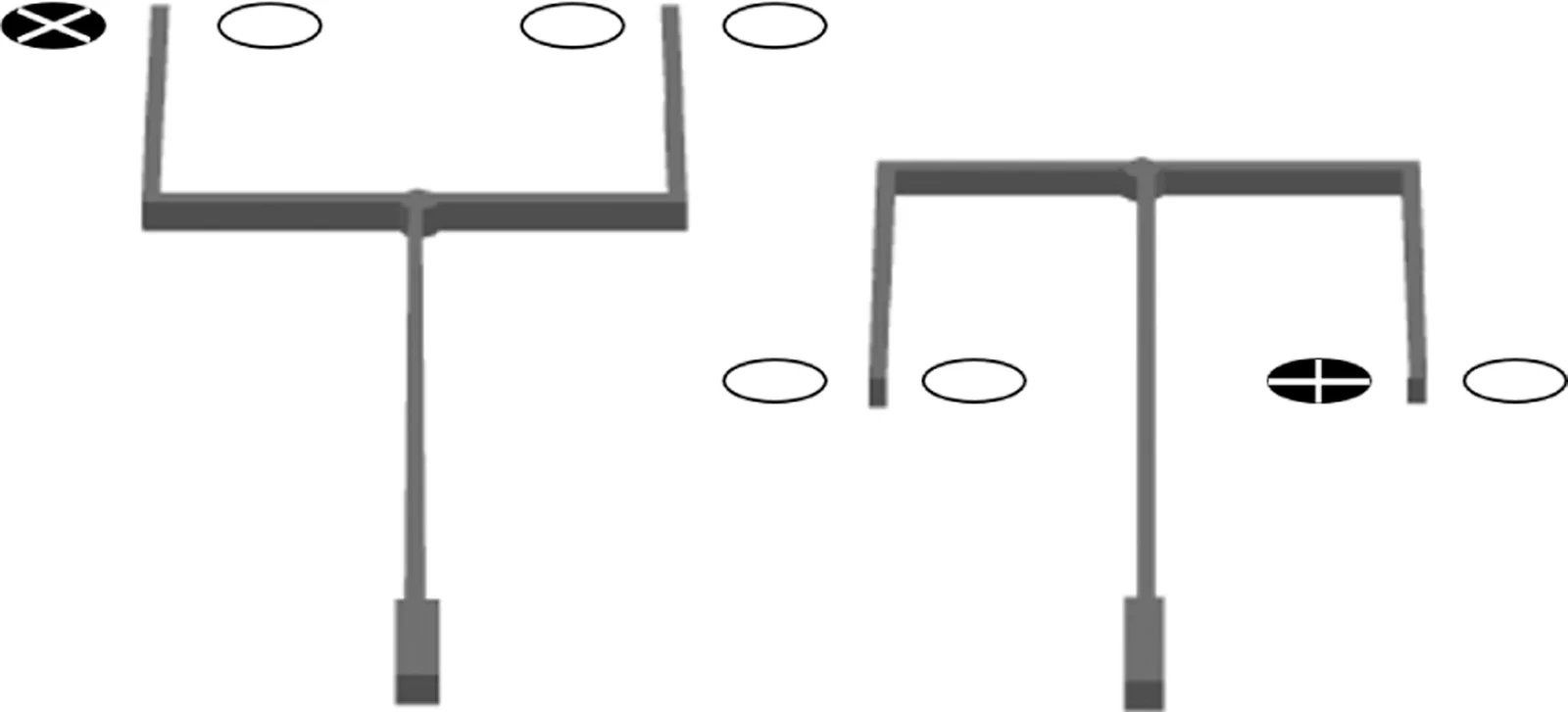
Stimuli and levers. *Note.* The U- and inverted-U lever are shown to the left and right, respectively. Each ellipse to the left and right of the levers’ effect points show the positions where each imperative stimuli might appear. The two filled ellipses show the “ × ” and “+” imperative stimuli. Note that only one imperative stimulus (and lever) was shown on each trial and the empty ellipses in this figure only show the potential positions of all stimuli, but were not presented in the experiment

#### Task and Procedure

The task was to manually press a response key to turn the lever either towards or away from the stimulus, depending on whether the stimulus was a “+” or “ × .” Participants were instructed to react as quickly as possible while trying to make as few mistakes as possible. They were told that pressing either the left or right key would result in a corresponding clockwise or counterclockwise turn of the lever, as if they were physically manipulating it.

At the beginning of each block, participants were presented with the current type of lever, which remained visible until the end of the respective block. Each trial started with the lever in the middle position, followed by stimulus onset after 1,500 ms. After a key press, the lever was immediately presented in its end position. The lever returned to the middle position after the key was released and a new trial began afterward.

Error feedback in the form of a tone (800 Hz) with a duration of 150 ms was given if the participant made an incorrect response, if an RT was shorter than 100 ms, or if no response was provided within 2,000 ms. The experiment lasted around 45 min, including self-paced breaks between blocks.

Participants performed 6 sequential blocks with each lever, and hence RE compatibility relation, with the order of levers counterbalanced across participants (a post-hoc exploratory analyses did not reveal any effect of RE compatibility order on the other reported results). Each block comprised 40 trials, resulting from 5 repetitions of all combinations of 4 locations × 2 stimulus types (“+” vs. “ × ”), presented in a random order within each block.

#### Design and Analysis

Trials were classified as *SR* compatible/incompatible, *SE* compatible/incompatible, and *RE* compatible/incompatible (see Fig. 2). Trials were SR compatible whenever the stimulus position and the response position matched, and incompatible otherwise. Trials were SE compatible whenever the movement direction of the effective part of the lever (i.e., its tip) matched the stimulus position, and incompatible otherwise. This essentially breaks down to trials with an imperative “+” stimulus being SE compatible, and trials with an imperative “ × ” stimulus being SE incompatible. Finally, trials with the U-lever were RE incompatible, and trials with the inverted-U lever were RE compatible.

Data were pre-processed in the following steps: Trials from the first block with either the U-shaped or inverted-U-shaped lever were discarded as practice. Subsequently, trials with RTs shorter than 100 ms, a time-out after 2,000 ms, or where participants performed more than one response were excluded (0.44%). We did not pre-register the procedure of excluding trials where participants performed more than one response. However, these occurred only 6 times in the test blocks.

For RT analyses, only correct trials were considered (discarding 8.51% of the trials). Following the original approach of Müsseler and Skottke (2011), median RTs were calculated for each participant and condition. An analogous analysis using means with an additional outlier exclusion based on *z*-values yielded the same conclusions. We thus only report the results with median RTs here, but interested readers can find files with the results using mean RTs in the OSF repository.

Median RTs and percent errors (PEs) were submitted to separate 2 × 2 × 2 repeated-measures Analyses of Variance (ANOVAs) with RE, SE, and SR compatibility (each compatible vs. incompatible) as factors. For both RTs and PEs, we followed up with 2 × 2 ANOVAs with the factors SE and SR compatibility, separately for each RE compatibility condition.

In our pre-registration, we also mentioned analyzing the duration of time participants pressed a key. However, apart from the insight that keys were pressed for a duration of about 160 ms and a statistically significant 7 ms effect between the levels of SE compatibility, nothing particularly insightful was gained from that analysis. We thus refrained from reporting this analysis in the main text, but interested readers can find the corresponding ANOVA output in the OSF repository.

### Results

#### RTs

Median RTs of the present replication are visualized in Figure 5A-B, where we have redrawn the results of the original study for ease of reference. The corresponding ANOVA indicated longer RTs for RE incompatible (723 ms) relative to compatible (654 ms) trials, *F*(1, 38) = 30.27, *p* < .001, $\eta_{p}^{2}=.44$ . Further, RTs were longer for SE incompatible (712 ms) relative to compatible (665 ms) trials, *F*(1, 38) = 71.19, *p* < .001, $\eta_{p}^{2}=.65$ . The main effect of SR compatibility did not reach statistical significance, *F*(1, 38) = 0.05, *p* = .823, $\eta_{p}^{2}<.01$ . The difference between SE incompatible and compatible trials was slightly larger for RE compatible (57 ms) relative to RE incompatible (36 ms) trials, *F*(1, 38) = 5.81, *p* = .021, $\eta_{p}^{2}=.13$ . Descriptively, responses were slower for SR incompatible relative to SR compatible trials with an incompatible RE relationship (4 ms), but this relationship reversed in the case of a compatible RE relationship (−11 ms), *F*(1, 38) = 3.72, *p* = .061, $\eta_{p}^{2}=.09$ . Furthermore, responses for SR incompatible trials were slower relative to SR compatible trials only for the SE compatible condition (26 ms). For SE incompatible conditions, in contrast, an incompatible SR relationship was advantageous (−28 ms), *F*(1, 38) = 30.51, *p* < .001, $\eta_{p}^{2}=.45$ . Importantly, this latter interaction of SR and SE compatibility was only present if the RE relationship was compatible, but not when it was incompatible, as suggested by the significant three-way interaction, *F*(1, 38) = 24.47, *p* < .001, $\eta_{p}^{2}=.39$ . Separate ANOVAs for each condition of RE compatibility supported this impression: The SR × SE interaction was significant for RE compatible trials, *F*(1, 38) = 50.31, *p* < .001, $\eta_{p}^{2}=.57$ , but not for RE incompatible trials, *F*(1, 38) = 0.01, *p* = .944, $\eta_{p}^{2}<.01$ .

**Figure 5.**
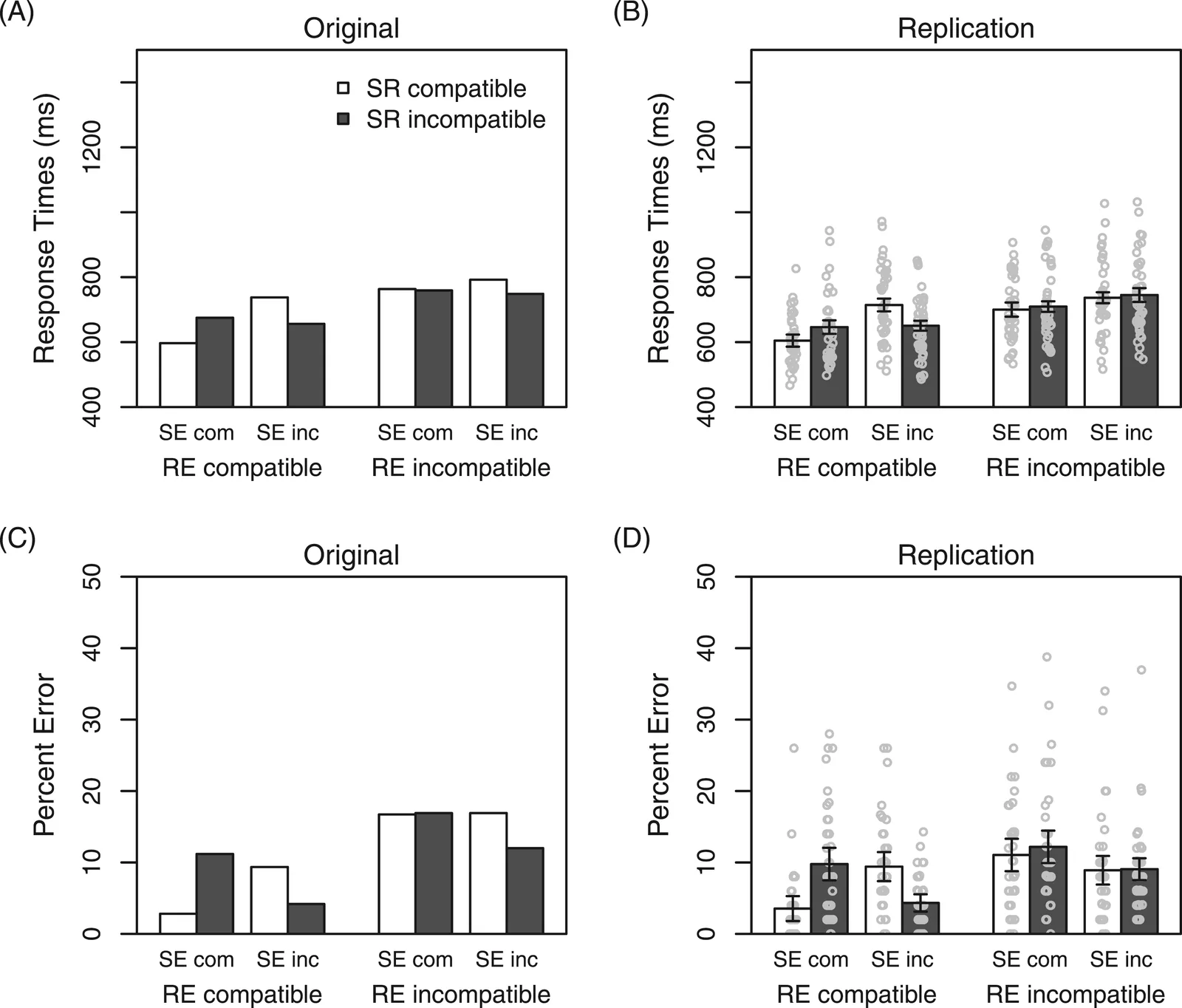
Median response times and percent errors for experiment 1. *Note*. Panels (A) and (B) show the results for RTs as a function of RE, SR, and SE compatibility, separately for the original and our replication in Experiment 1. Panels (C) and (D) show the analogues results for PEs. White and dark gray bars represent compatible and incompatible SR relationships, respectively. In the panels for our replication study, gray circles indicate individual observations. Note that Panels A and C are identical to Figure 3 and just redrawn for reference. Com = compatible; inc = incompatible. Error bars indicate 95% confidence intervals after removing between-participant variance (Morey, 2008)

#### PEs

PEs of the present replication are visualized in Figure 5C-D, where we have redrawn the results of the original study for ease of reference. The corresponding ANOVA indicated higher PEs for RE incompatible (10.3%) relative to compatible (6.8%) trials, *F*(1, 38) = 10.24, *p* = .003, $\eta_{p}^{2}=.21$ . Surprisingly, PEs were higher for SE compatible (9.1%) relative to incompatible (7.9%) trials, *F*(1, 38) = 6.53, *p* = .015, $\eta_{p}^{2}=.15$ . The main effect of SR compatibility did not reach statistical significance, *F*(1, 38) = 1.30, *p* = .261, $\eta_{p}^{2}=.03$ . The difference in percent points between SE compatible and incompatible trials was slightly larger for RE incompatible (−2.6%) relative to RE compatible (0.2%) trials, *F*(1, 38) = 7.33, *p* = .010, $\eta_{p}^{2}=.16$ . The two-way interaction between RE and SR compatibility was not significant, *F*(1, 38) = 0.01, *p* = .944, $\eta_{p}^{2}<.01$ . Mirroring the RT results, responses for SR incompatible trials were more error prone relative to SR compatible trials only for the SE compatible condition (3.7%). For SE incompatible conditions, however, an incompatible SR relationship was advantageous (−2.5%), *F*(1, 38) = 17.15, *p* < .001, $\eta_{p}^{2}=.31$ . Importantly, this last interaction between SR and SE compatibility was only present if the RE relationship was compatible, but not when it was incompatible, as indicated by the significant three-way interaction, *F*(1, 38) = 14.59, *p* < .001, $\eta_{p}^{2}=.28$ . Separate ANOVAs for each condition of RE compatibility supported this impression: The SR × SE interaction was significant for RE compatible trials, *F*(1, 38) = 49.74, *p* < .001, $\eta_{p}^{2}=.57$ , but not for RE incompatible trials, *F*(1, 38) = 0.18, *p* = .677, $\eta_{p}^{2}<.01$ .

### Discussion

Experiment 1 replicated the experiment reported by Müsseler and Skottke (2011), and the results are straightforward. A glance at Figure 5 quickly reveals that the current pattern of results closely matches the original one. The most important finding is the presence of the SR × SE interaction for RE compatible trials, but not for RE incompatible trials. Given this, we are confident that the absent interaction in Müsseler and Skottke (2011) is not a false negative that might be attributed to a low sample size.

This (successful) replication served as a first step for the extensions of the design in the subsequent Experiments 2–4. To reiterate, our goal was to investigate the stability of the three-way interaction by various manipulations that likely affect the strength of RE compatibility effects (see Table 1). For instance, it is unclear whether the three-way interaction is primarily driven by the discrete/abrupt movement of the lever, which leads to a salience of the anticipated RE association (Exp. 2). Furthermore, previous studies have shown that the RE compatibility effects increase with the dimensional overlap between responses and effects (Exp. 3) and that they decrease with touchless gestures (Exp. 4).

## Experiment 2

Experiment 2 provides the first extension of the original study by Müsseler and Skottke (2011) and serves as a conceptual replication of that study and our Experiment 1. The main difference from these experiments is that the lever movement was not discrete, but instead the lever rotated continuously as long as the corresponding response button was pressed.

### Method

#### Transparency and Openness

All data, analyses scripts, experimental source code, and stimulus materials are available on OSF (Koob & Janczyk, 2025). Data were analyzed using the same software as in Experiment 1 (see https://aspredicted.org/my88-8694.pdf for the pre-registration). All procedures were in accordance with the Declaration of Helsinki.

#### Participants

We obtained data from students at the University of Bremen in 2023 until *n* = 40 valid data sets were collected. Three participants with more than 20% erroneous trials (after excluding training) were replaced. The final sample consisted of 30 female and 10 male students, ranging in age from 20 to 39 years (*M*_*age*_ = 25.4). After an initial visual inspection of the data, we excluded five participants whose RT or PE data deviated from the remaining participants in at least one condition. Again, including or excluding these data did not change our overall conclusions; therefore, we report the results without them (the results with all individuals can be found in the repository). All participants received course credit or monetary compensation, provided written informed consent, and had normal or corrected-to-normal vision.

#### Apparatus, Stimuli, Task, and Procedure

The apparatus and task were identical to those in Experiment 1. The trial procedure was also similar, with three key differences: First, upon a key press, the lever now rotated continuously in a clockwise or counterclockwise direction as long as the respective key was held down. To enable smooth lever movement, we interpolated the original three lever images into 65 frames using the open-source program Flowframes v. 1.36.0. The resulting stimulus material is available in the OSF repository. Second, upon reaching the end position, the lever jumped back to the center position to indicate that it had been turned sufficiently, either immediately upon reaching the stimulus or after the 150 ms error beep tone. The next trial began once all keys were released, following an inter-trial interval (ITI) of 1,500 ms with target stimulus onset. Third, due to the time required for continuous lever rotation, we extended the time-out of a trial to 4,000 ms.

#### Design and Analysis

As in Experiment 1, trials were classified as *RE* compatible/incompatible, *SE* compatible/incompatible, and *SR* compatible/incompatible (see Figure 2).

Data were pre-processed in the following steps: Trials from the first block with either the U-shaped or inverted-U-shaped lever were discarded as practice. Afterward, trials with RTs shorter than 100 ms or a time-out after 4,000 ms (0.34%) were excluded. Since a continuous lever movement allows participants to correct initially erroneous responses, we analyzed (1) the percent of trials with an initially erroneous response (PIEs), (2) the percent of trials where participants ended in an erroneous position (PEs), and (3) the percent of initially erroneous responses that were changed/corrected and the lever ended in the correct end position (PChs). The initiation time for the movement (i.e., the time-point of the first button press) was considered as the RT.

For RT analyses, only those trials were considered where participants responded correctly initially, did not correct their response during the movement, and ended in the correct end position (discarding 17.07% of the trials). Similar ANOVAs were conducted as in Experiment 1.

### Results

#### RTs

Median RTs are visualized in Figure 6A. The corresponding ANOVA indicated longer RTs for RE incompatible (734 ms) relative to compatible (671 ms) trials, *F*(1, 34) = 5.30, *p* = .028, $\eta_{p}^{2}=.13$ . Further, RTs were longer for SE incompatible (730 ms) relative to compatible (676 ms) trials, *F*(1, 34) = 46.50, *p* < .001, $\eta_{p}^{2}=.58$ . The main effect of SR compatibility did not reach statistical significance, *F*(1, 34) = 2.69, *p* = .110, $\eta_{p}^{2}=.07$ . The difference between SE incompatible and compatible trials was slightly larger for RE compatible (71 ms) relative to RE incompatible (37 ms) trials, *F*(1, 34) = 8.29, *p* = .007, $\eta_{p}^{2}=.20$ . Responses were slightly slower for SR incompatible relative to SR compatible conditions in case the RE relationship was incompatible (3 ms), but faster when the RE relationship was compatible (−22 ms), *F*(1, 34) = 7.76, *p* = .009, $\eta_{p}^{2}=.19$ . Furthermore, responses for SR incompatible trials were slower relative to SR compatible trials only for the SE compatible condition (20 ms). For SE incompatible conditions, in contrast, an incompatible SR relationship was advantageous (−38 ms), *F*(1, 34) = 22.57, *p* < .001, $\eta_{p}^{2}=.40$ . Importantly, this latter interaction of SR and SE compatibility was only present if the RE relationship was compatible, but not when it was incompatible, as suggested by a significant three-way interaction, *F*(1, 34) = 23.36, *p* < .001, $\eta_{p}^{2}=.41$ . Separate ANOVAs for each condition of RE compatibility supported this impression: The SR × SE interaction was significant for RE compatible trials, *F*(1, 34) = 40.60, *p* < .001, $\eta_{p}^{2}=.54$ , but not for RE incompatible trials, *F*(1, 34) < 0.01, *p* = .963, $\eta_{p}^{2}<.01$ .

**Figure 6.**
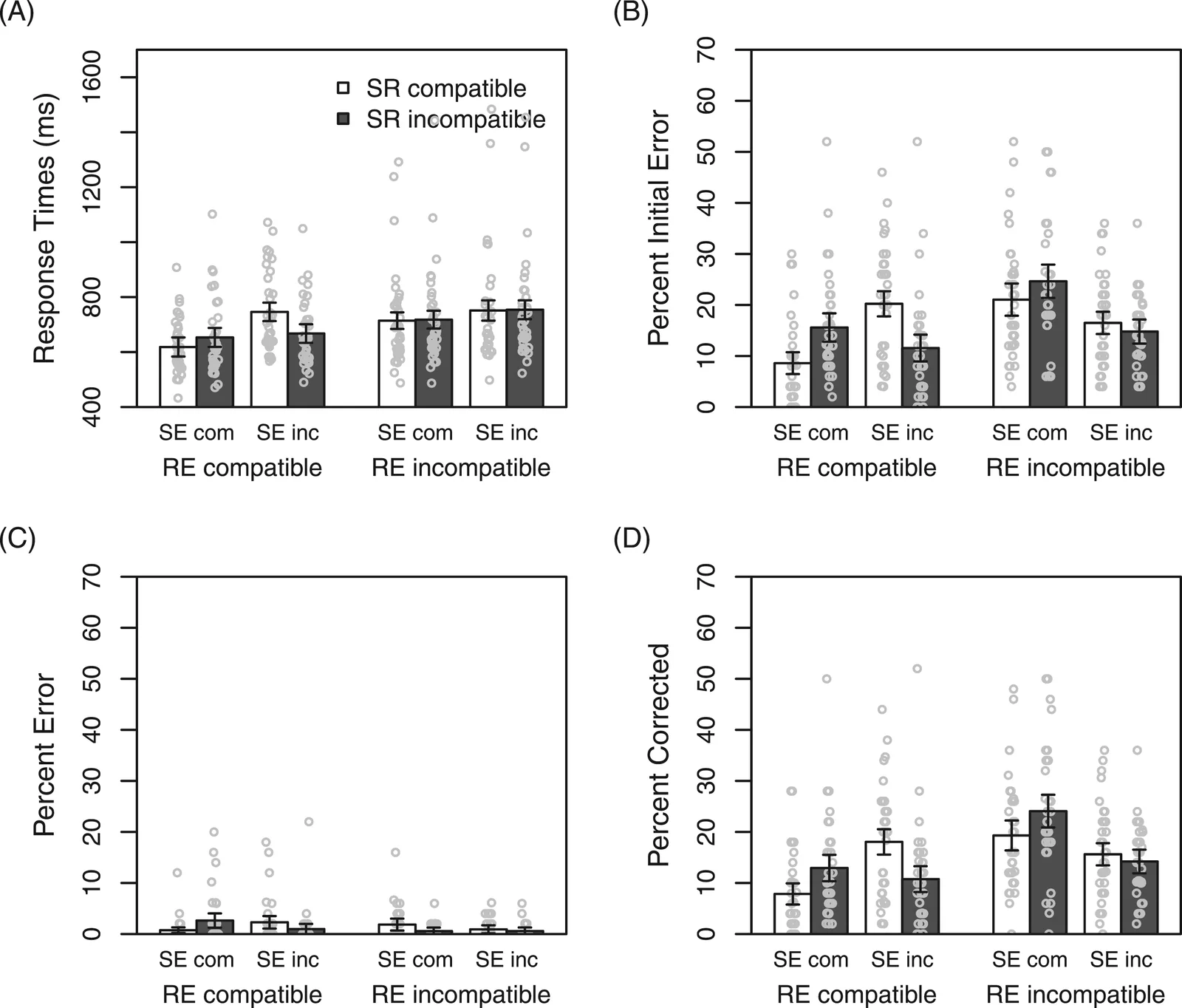
Median response time and percent of initial erroneous responses, error responses, and corrected responses for Experiment 2. *Note.* Panel (A) shows the results for RTs as a function of RE, SR, and SE compatibility, similar to Figure 5. Panels (B), (C), and (D) show the analogues results for the percent of initially erroneous, erroneous, and corrected responses, respectively. White and dark gray bars represent compatible and incompatible SR relationships, respectively. Gray circles indicate individual observations. Error bars indicate 95% confidence intervals after removing between-participant variance (Morey, 2008)

#### PIEs

Mean PIEs are visualized in Figure 6B. The corresponding ANOVA indicated higher PIEs for RE incompatible (19.2%) relative to compatible (14.0%) trials, *F*(1, 34) = 11.17, *p* = .002, $\eta_{p}^{2}=.25$ . PIEs were higher for SE compatible (17.5%) relative to incompatible (15.8%) trials, *F*(1, 34) = 6.74, *p* = .014, $\eta_{p}^{2}=.17$ . The main effect of SR compatibility did not reach statistical significance, *F*(1, 34) < 0.01, *p* = .946, $\eta_{p}^{2}<.01$ . The difference between SE compatible and incompatible trials was larger for RE incompatible (−7.2%) relative to RE compatible (3.8%) trials, *F*(1, 34) = 35.72, *p* < .001, $\eta_{p}^{2}=.51$ . The two-way interaction between RE and SR compatibility did not reach statistical significance, *F*(1, 34) = 2.37, *p* = .133, $\eta_{p}^{2}=.07$ . Responses for SR incompatible trials were more likely to yield initially erroneous responses relative to SR compatible trials only for the SE compatible condition (5.3%). For SE incompatible conditions, however, an incompatible SR relationship was advantageous (−5.2%), *F*(1, 34) = 42.57, *p* < .001, $\eta_{p}^{2}=.56$ . Importantly, this latter interaction between SR and SE compatibility was present primarily for the RE compatible relationship. For the RE incompatible relationship, the SE × SR interaction was qualitatively similar, but less pronounced, as indicated by a significant three-way interaction, *F*(1, 34) = 10.91, *p* = .002, $\eta_{p}^{2}=.24$ . Separate ANOVAs for each condition of RE compatibility supported this impression, although both ANOVAs yielded a statistically significant two-way interaction between SE × SR compatibility; RE compatible trials: *F*(1, 34) = 59.13, *p* < .001, $\eta_{p}^{2}=.63$ ; and RE incompatible trials: *F*(1, 34) = 4.74, *p* = .036, $\eta_{p}^{2}=.12$ .

#### PEs

Mean PEs are visualized in Figure 6C. Overall, PEs were low, and we thus report the results in a shortened form. The corresponding ANOVA indicated a two-way interaction between RE and SR compatibility, *F*(1, 34) = 8.62, *p* = .006, $\eta_{p}^{2}=.20$ . Responses for SR incompatible trials were more error prone relative to SR compatible trials for RE compatible conditions (0.3%). For RE incompatible conditions, an SR incompatible relationship was more advantageous (−0.8%). Again, we observed a significant three-way interaction, *F*(1, 34) = 9.27, *p* = .004, $\eta_{p}^{2}=.21$ . No other main or interaction effect reached statistical significance. Separate ANOVAs for each condition of RE compatibility yielded a statistically significant two-way interaction between SE × SR compatibility only for RE compatible relationships, RE compatible trials: *F*(1, 34) = 5.48, *p* = .025, $\eta_{p}^{2}=.14$ ; RE incompatible trials: *F*(1, 34) = 2.66, *p* = .112, $\eta_{p}^{2}=.07$ .

#### PChs

Mean PChs are visualized in Figure 6D and closely followed the pattern of PIEs. The corresponding ANOVA indicated higher PChs for RE incompatible (18.3%) relative to compatible (12.4%) trials, *F*(1, 34) = 14.47, *p* = .001, $\eta_{p}^{2}=.30$ . PChs were higher for SE compatible (16.0%) relative to incompatible (14.7%) trials, *F*(1, 34) = 4.85, *p* = .034, $\eta_{p}^{2}=.12$ . The main effect of SR compatibility was not significant, *F*(1, 34) = 0.15, *p* = .703, $\eta_{p}^{2}<.01$ . SE compatible trials were more often corrected than SE incompatible trials for RE incompatible (−6.8%) relative to RE compatible (4.0%) trials, *F*(1, 34) = 42.69, *p* < .001, $\eta_{p}^{2}=.56$ . The two-way interaction between SR and RE compatibility was significant, *F*(1, 34) = 6.64, *p* = .014, $\eta_{p}^{2}=.16$ . Responses for SR compatible trials were more likely to yield a corrected response relative to SR incompatible trials for the RE compatible condition (−1.1%). For RE incompatible conditions, the pattern was reversed (1.6%). Furthermore, we again observed a significant SE × SR interaction, *F*(1, 34) = 29.58, *p* < .001, $\eta_{p}^{2}=.47$ . As with the PIEs and RTs, PChs were higher for SR incompatible relative to compatible trials only for the SE compatible condition (4.9%). For SE incompatible conditions, an incompatible SR relationship was advantageous (−4.3%). This interaction between SR and SE compatibility was again stronger for the RE compatible relative to the RE incompatible relationship, as indicated by a significant three-way interaction, *F*(1, 34) = 4.76, *p* = .036, $\eta_{p}^{2}=.12$ . Separate ANOVAs for each condition of RE compatibility supported this impression, although both ANOVAs yielded a statistically significant two-way interaction between SE × SR compatibility, RE compatible trials: *F*(1, 34) = 42.96, *p* < .001, $\eta_{p}^{2}=.56$ ; RE incompatible trials: *F*(1, 34) = 6.07, *p* = .019, $\eta_{p}^{2}=.15$ .

### Discussion

The results of Experiment 2 generally align with those of Experiment 1. In particular, the two-way interaction between SE and SR compatibility was more pronounced in RE compatible trials compared to RE incompatible trials. For RTs and PEs (see Figure 8A and C), this interaction was not observed in RE incompatible trials, thus replicating the results of Experiment 1 and the original experiment of Müsseler and Skottke (2011). However, for PIEs and PChs, the SE × SR interaction reached statistical significance even in RE incompatible trials, although it was smaller, yet qualitatively similar, to the interaction in RE compatible trials.

In sum, Experiments 1 and 2 show that the different interaction between SR and SE compatibility depending on the level of RE compatibility remains relatively stable with regard to RTs and PEs, even when participants perform a continuous rotation of the lever using key presses. However, they also suggest that the interaction between SR and SE compatibility may be present even in RE incompatible trials, as indicated by the results of Experiment 2 for PIEs and PChs. Thus, conclusions regarding the interaction between SR and SE compatibility may depend on the dependent variable used.

## Experiment 3

Experiment 3 addressed the extent to which the previous results might change when the dimensional overlap between the effects and the response input is increased. To this end, participants controlled the lever with a slider, and both the lever movement on the screen and the response input were continuous.

### Method

#### Transparency and Openness

All data, analyses scripts, experimental source code, and stimulus materials are available on OSF (Koob & Janczyk, 2025). The data were analyzed using the same software as described for Experiment 1 (see https://aspredicted.org/nkv4-kgvb.pdf for the pre-registration). All procedures were in accordance with the Declaration of Helsinki.

#### Participants

We obtained data from students at the University of Bremen until *n* = 40 valid data sets were collected. During the sampling process in 2023, one participant with more than 20% erroneous trials after excluding training was replaced. The final sample comprised 33 female and 7 male students, aged between 19 and 42 years (*M*_*age*_ = 25.4). After an initial visual data inspection, we excluded one participant whose RT data clearly deviated from the remaining participants in at least one condition. Including or excluding this participant’s data did not change the overall conclusions, and the results are reported without these data (the OSF repository contains the results with all participants). All participants received course credit or monetary compensation, provided written informed consent, and had normal or corrected-to-normal vision.

#### Apparatus, Stimuli, Task, and Procedure

The task and stimuli were identical to those in Experiment 2. However, in contrast to the previous study, participants now operated a slider placed in front of them (see Figure 7A for an illustration). This slider was directly coupled with the lever on the screen and was locked and unlocked by the experimental software (see the next paragraph). When participants moved the slider to the left or right, the lever continuously rotated clockwise or counterclockwise, respectively, as a direct function of this movement. Thus, participants could now directly control the lever’s movements.

**Figure 7.**
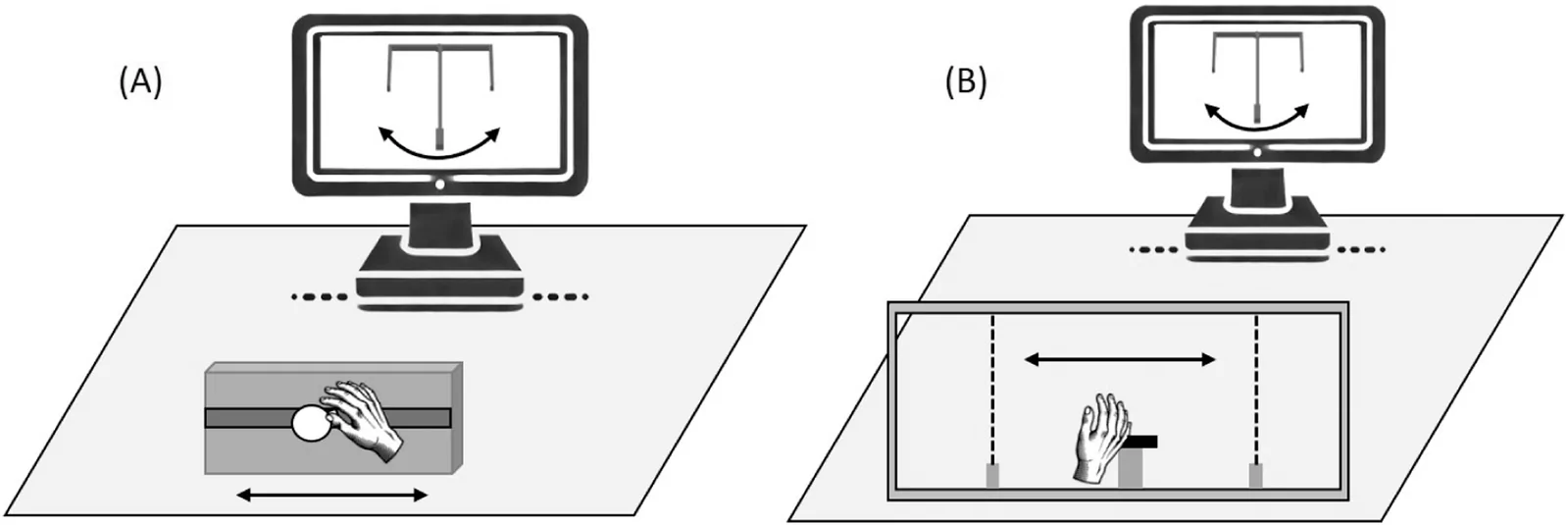
Illustration of the apparatus in Experiment 3 and 4. *Note*. Panel (A) shows the apparatus for Experiment 3. Participants controlled the lever using a slider placed in front of them on a table (with the knob, where participants grasped the slider, pointing towards the participants). Moving the slider to the left or right resulted in continuous clockwise or counterclockwise rotations of the lever, as if participants were controlling the handle. Panel (B) shows the apparatus for Experiment 4. Participants controlled the lever using touchless gestures. At the beginning of the trial, participants placed their small finger on a touch-sensitive key. Gestures were detected when the hand crossed one of two light barriers (the black dashed lines), causing the lever to rotate (gesture to the left for clockwise rotation; gesture to the right for counterclockwise rotation). The touch-sensitive key and the light barriers were mounted inside a wooden frame (75 cm × 27 cm)

The trial procedure was similar to the previous experiments, with some modifications. A trial began with the presentation of the lever in the center position. After 150 ms, the target stimulus appeared, and the slider was unlocked. Participants then moved the slider until the lever had rotated to a sufficient degree, as indicated by the slider being locked and becoming unmovable. The slider remained locked for 1,000 ms (with an additional 150 ms if an error beep tone occurred). Afterward, the target stimulus and the lever image were removed from the screen, and participants moved the slider back to the center position. Once the center position was reached, the slider was locked again, and the next trial began after an ITI of 1,500 ms.

#### Design and Analysis

Trials were classified as *RE* compatible/incompatible, *SE* compatible/incompatible, and *SR* compatible/incompatible (see Figure 2). Data were pre-processed in the following steps: Trials from the first block with either a U-shaped or inverted-U-shaped lever were discarded as practice. Next, trials with a response initiation faster than 100 ms or a time-out after 4,000 ms (1.29%) were excluded. Similar to Experiment 2, we analyzed PIEs, PEs, PChs, and RTs. For RTs, we considered the initiation time, measured from stimulus onset until participants moved the slider to the left or right of the center across a specified boundary. These boundaries were 5% of the total movement range to the left and right of the center position of the slider. A response was classified as “corrected/changed” if participants initially crossed a boundary to the left or right, but then moved the slider in the opposite direction, crossing the other boundary.

For RT analyses, only trials in which participants made an initially correct response, did not correct their response during the movement, and ended in a correct final position were included (discarding 8.44% of the trials). The following ANOVAs were conducted as in Experiment 1.

### Results

#### RTs

Median RTs are visualized in Figure 8A. The corresponding ANOVA indicated longer RTs for RE incompatible (762 ms) relative to compatible (675 ms) trials, *F*(1, 38) = 145.75, *p* < .001, $\eta_{p}^{2}=.79$ . Further, RTs were longer for SE incompatible (740 ms) relative to compatible (697 ms) trials, *F*(1, 38) = 96.76, *p* < .001, $\eta_{p}^{2}=.72$ . The main effect of SR compatibility did not reach statistical significance, *F*(1, 38) = 1.73, *p* = .196, $\eta_{p}^{2}=.04$ . In contrast to the previous experiments, none of the two-way interactions turned out statistically significant, SE × RE compatibility: *F*(1, 38) = 1.23, *p* = .274, $\eta_{p}^{2}=.03$ ; SR × RE compatibility: *F*(1, 38) = 0.03, *p* = .872, $\eta_{p}^{2}<.01$ ; SE × SR compatibility: *F*(1, 38) = 0.57, *p* = .454, $\eta_{p}^{2}=.01$ . Yet, again we observed a three-way interaction, *F*(1, 38) = 78.07, *p* < .001, $\eta_{p}^{2}=.67$ . In contrast to the previous experiments, however, where the three-way interaction in RTs indicated a reduced SE × SR interaction in RE incompatible relative to compatible trials, the respective interaction was clearly present for both RE compatibility conditions, although in opposite directions. For RE compatible trials, we observed the SR incompatible condition to be harmful for performance in SE compatible conditions (35 ms), but advantageous in SE incompatible conditions (−41 ms). The reverse was true for RE incompatible trials. Here, we observed the SR compatible condition to be harmful for performance in SE compatible conditions (−49 ms), but advantageous in SE incompatible conditions (40 ms). The separate ANOVAs for each condition of RE compatibility yielded both significant two-way interactions, SR × SE interaction for RE compatible trials: *F*(1, 38) = 66.87, *p* < .001, $\eta_{p}^{2}=.64$ ; for RE incompatible trials: *F*(1, 38) = 34.03, *p* < .001, $\eta_{p}^{2}=.47$ .

**Figure 8.**
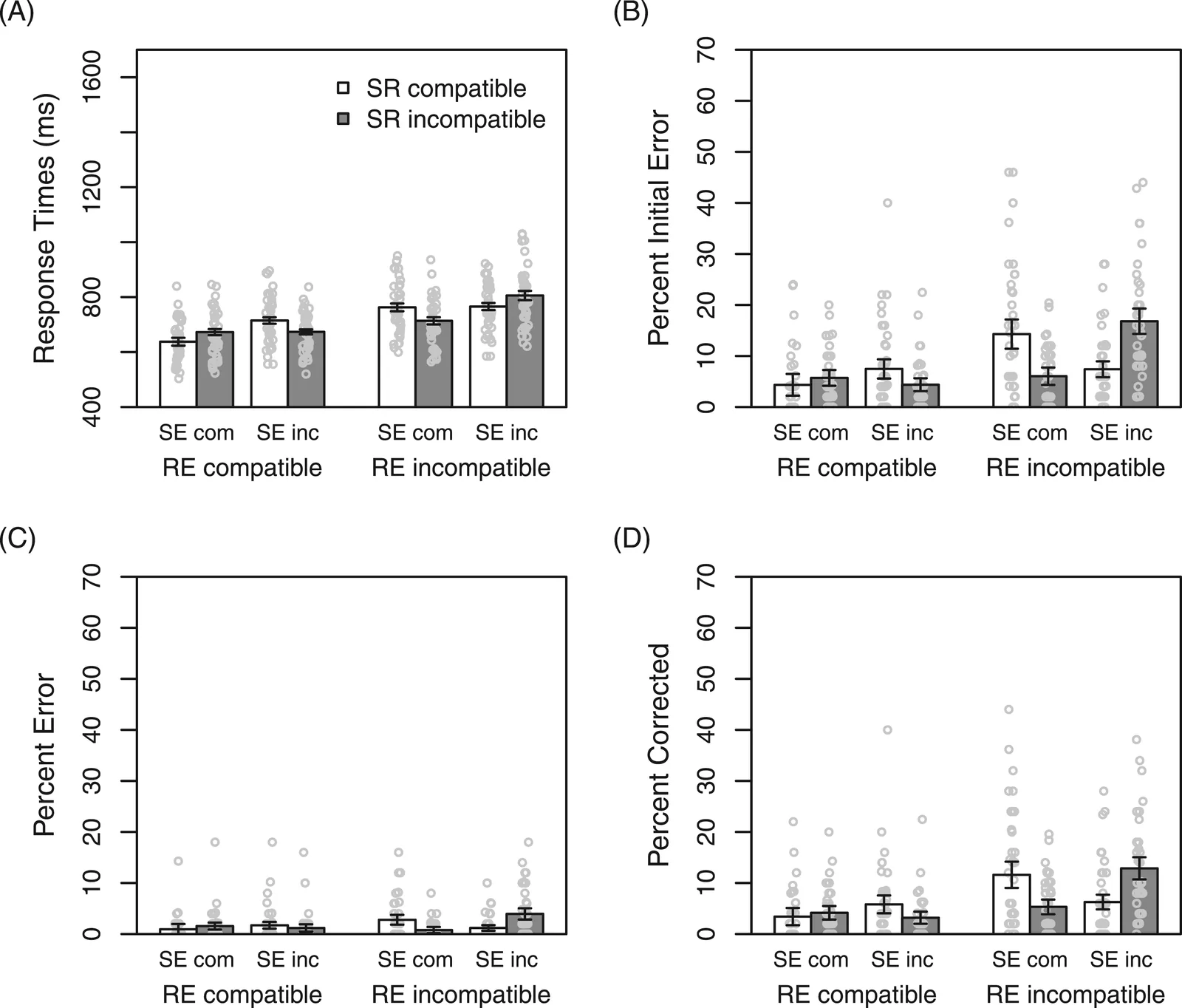
Median response time and percent of initial erroneous, error, and corrected responses for Experiment 3. *Note.* Panel (A) shows the results for RTs as a function of RE, SR, and SE compatibility. Panels (B), (C), and (D) show the analogues results for percents initial erroneous, error, and corrected responses, respectively. White and dark gray bars represent compatible and incompatible SR relationships, respectively. Gray circles indicate individual observations. Error bars indicate 95% confidence intervals after removing between-participant variance (Morey, 2008)

#### PIEs

Mean PIEs are visualized in Figure 8B. The corresponding ANOVA indicated higher PIEs for RE incompatible (11.1%) relative to compatible (5.5%) trials, *F*(1, 38) = 40.83, *p* < .001, $\eta_{p}^{2}=.52$ . PIEs were higher for SE incompatible (9.0%) relative to compatible (7.6%) trials, *F*(1, 38) = 5.47, *p* = .025, $\eta_{p}^{2}=.13$ . The main effect of SR compatibility did not reach statistical significance, *F*(1, 38) = 0.07, *p* = .793, $\eta_{p}^{2}<.01$ . The two-way interactions between RE and SE compatibility, $F\left(1,38\right)=0.66,p=.422,\eta_{p}^{2}=.02$ , and between RE and SR compatibility, $F\left(1,38\right)=1.00,p=.324,\eta_{p}^{2}=.03$ , were not significant. Yet, the interaction between SE and SR compatibility reached statistical significance, *F*(1, 38) = 24.08, *p* < .001, $\eta_{p}^{2}=.39$ . Responses for SR incompatible trials were more likely to yield initial erroneous responses relative to SR compatible trials for the SE incompatible condition (3.2%). For SE compatible conditions, an incompatible SR relationship was advantageous (−3.4%). Importantly, this interaction between SR and SE compatibility was again modulated by RE compatibility, *F*(1, 38) = 54.48, *p* < .001, $\eta_{p}^{2}=.59$ . For RE compatible trials, we observed the SR incompatible condition to be slightly more harmful for performance in SE compatible conditions (1.4 %), but advantageous in SE incompatible conditions (−3.1%). The reverse was true for RE incompatible trials. Here, we observed the SR compatible condition to be harmful for performance in SE compatible conditions (−8.3%), but advantageous in SE incompatible conditions (9.4%). Separate ANOVAs for each condition of RE compatibility yielded statistically significant two-way interactions between SE × SR compatibility, for RE compatible trials: *F*(1, 38) = 9.91, *p* = .003, $\eta_{p}^{2}=.21$ ; for RE incompatible trials: *F*(1, 38) = 51.09, *p* < .001, $\eta_{p}^{2}=.57$ .

#### PEs

Mean PEs are visualized in Figure 8C. Overall, PEs were low, and we thus report the results in a shortened form. The corresponding ANOVA indicated a statistically significant main effect of RE compatibility, *F*(1, 38) = 9.25, *p* = .004, $\eta_{p}^{2}=.20$ . Responses were more error prone on RE incompatible (2.2%) relative to compatible (1.3%) trials. Furthermore, a two-way interaction between SE and SR compatibility emerged, *F*(1, 38) = 6.86, *p* = .013, $\eta_{p}^{2}=.15$ . Responses for SR incompatible trials were more error prone relative to SR compatible trials for the SE incompatible condition (1.1%). For SE compatible conditions, an incompatible SR relationship was advantageous (−0.7%). Furthermore, we again observed a significant three-way interaction, *F*(1, 38) = 16.92, *p* < .001, $\eta_{p}^{2}=.31$ . No other main or interaction effect turned out statistically significant. Separate ANOVAs for each condition of RE compatibility yielded a statistically significant two-way interaction only for RE incompatible relationships, for RE compatible trials: *F*(1, 38) = 1.74, *p* = .195, $\eta_{p}^{2}=.04$ ; for RE incompatible trials: *F*(1, 38) = 18.38, *p* < .001, $\eta_{p}^{2}=.33$ .

#### PChs

Mean PChs are visualized in Figure 8D, following the pattern of PIEs. The corresponding ANOVA indicated higher PChs for RE incompatible (9.0%) relative to compatible (4.1%) trials, *F*(1, 38) = 33.23, *p* < .001, $\eta_{p}^{2}=.47$ . The main effects of SE and SR compatibility did not reach statistical significance, *F*(1, 38) = 3.09, *p* = .087, $\eta_{p}^{2}=.08$ and *F*(1, 38) = 0.61, *p* = .441, $\eta_{p}^{2}=.02$ , respectively. The two-way interactions between RE and SE compatibility, $F\left(1,38\right)=0.11,p=.739,\eta_{p}^{2}<.01$ , and between RE and SR compatibility, $F\left(1,38\right)=0.82,p=.370,\eta_{p}^{2}=.02$ , were not significant. Yet, we observed a significant two-way interaction between SE and SR compatibility, *F*(1, 38) = 21.31, *p* < .001, $\eta_{p}^{2}=.36$ . PChs were lower for SR incompatible relative to compatible trials for the SE compatible condition (−2.8%). For SE incompatible conditions, a compatible SR relationship was advantageous (2.0%). Importantly, however, we again observed a statistically significant three-way interaction, *F*(1, 38) = 41.26, *p* < .001, $\eta_{p}^{2}=.52$ , indicating that the SE × SR interaction was clearly present for both RE compatibility conditions, although in opposite directions. For RE compatible trials, we observed more response corrections for SR incompatible relative to compatible conditions in SE compatible conditions (0.8%), but less error corrections in SE incompatible conditions (−2.6%). The reverse was true for RE incompatible trials. Here, we observed more response corrections for SR compatible relative to incompatible conditions in SE compatible conditions (−6.3%), but less response corrections for SE incompatible conditions (6.6%). The separate ANOVAs for each condition of RE compatibility yielded both significant two-way interactions, SR × SE interaction for RE compatible trials: *F*(1, 38) = 8.06, *p* = .007, $\eta_{p}^{2}=.17$ ; SR × SE interaction for RE incompatible trials: *F*(1, 38) = 42.34, *p* < .001, $\eta_{p}^{2}=.53$ .

### Discussion

The results of Experiment 3 resemble but also differ from those obtained in Experiments 1 and 2. First, in the RE compatible condition, the interaction of SR and SE compatibility was in the direction as observed in the previous experiments. Secondly, however, we observed a significant interaction of SE × SR in the RE incompatible condition as well. Interestingly, while we also observed an interaction of SE × SR in the RE incompatible condition in Experiment 2 for PIEs and PChs, this interaction turned out opposite in Experiment 3. Specifically, in RE compatible conditions, performance was best when SE and SR compatibility matched, whereas in RE incompatible conditions, performance was best when SE and SR compatibility mismatched. Furthermore, this pattern was observed for all dependent variables. Although the interaction of SE × SR in RE incompatible trials were different for Experiment 3 and Experiment 2, it is at least fair to say that an RE incompatible relationship does not simply override the interaction of SE and SR compatibility, but rather modulates it, particularly once the dimensional overlap between responses and stimuli is increased. Experiment 4 will implement a response that had a negative impact on the RE compatibility effect in previous studies.

## Experiment 4

Experiment 4 investigated how the SE × SR compatibility interaction manifests with another input device, namely, touchless gestures. These movements may be special, as previous research did not consistently provide evidence for an RE compatibility effect with touchless gestures (Janczyk, 2023; Janczyk et al., 2019): While SR compatibility exerted large effects in these studies, RE compatibility was not observed or, if anything, the effect was only very small in size.

The most straightforward prediction in this case is that we expect no main effect of RE compatibility, but rather the presence of main effects of SR and SE compatibility. The extent to which these factors interact, however, is less clear, as no previous study has jointly considered SR, RE, and SE compatibility with touchless gestures, while clear effects of SR compatibility have been observed in previous studies with touchless gestures. A preliminary conjecture is that the interaction of SE and SR compatibility remains consistent across both RE compatible and RE incompatible conditions.

### Method

#### Transparency and Openness

All data, analyses scripts, experimental source code, and stimulus materials are available on OSF (Koob & Janczyk, 2025). Data were analyzed with the same software as described in Experiment 1 (see https://aspredicted.org/6wmp-9mv9.pdf for the pre-registration). All procedures were in accordance with the Declaration of Helsinki.

#### Participants

We obtained data from students at the University of Bremen until *n* = 40 valid data sets were collected. During the sampling process in 2023–2024, 16 participants with more than 20% erroneous trials after excluding training were replaced. The final sample comprised 31 female and 9 male students, ranging in age from 17 to 31 years (*M*_*age*_ = 22.02). After an initial visual data inspection, we excluded 1 participant for yielding PE data that deviated from the remaining participants in one condition. Including or excluding this individual did not change our overall conclusions, so we report the results after excluding this participant for brevity (the OSF repository contains the results with all participants). All participants received course credit or monetary compensation, provided written informed consent, and had normal or corrected-to-normal vision.

#### Apparatus, Stimuli, Task, and Procedure

The task and stimuli were identical to Experiment 1. However, participants now operated the lever using touchless gestures. The response apparatus consisted of a central touch-sensitive key and two light barriers positioned to the left and right of the central key (see Figure 7B; the distance between each light barrier and the central key was approximately 7 cm). Participants were instructed to place their little finger on a touch-sensitive key, with their fingers outstretched and their thumb pointing upward. To control the lever, participants had to lift their hand from the touch-sensitive key and move it rapidly to the left or right. While doing so, they should flex or extend their hand at the wrist, depending on the direction of movement (e.g., right-handed participants flexed their hand when moving it to the left). The direction of this “gesture” to the left or right was measured by light barriers and resulted in a clockwise or counterclockwise rotation of the lever, as if controlling the handle of the lever. The experimenter instructed the participants carefully with respect to the correct hand movement and corrected them whenever necessary. RTs were defined as the time from stimulus onset until the hand left the touch-sensitive key.

The trial procedure was similar to Experiment 1, with some modifications. The trial started with the presentation of a lever in the center position, followed by the target stimulus after 1,500 ms. After a gesture to the left or right, the lever was immediately presented in its corresponding end position for 160 ms before jumping back to its central position. The duration of 160 ms was based on the average key press duration in Experiment 1. Once the participant returned their hand to the central touch-sensitive key, the next trial began.

Similar to the previous experiments, error feedback in the form of a tone was provided if the participant made an incorrect response, if the RT was shorter than 100 ms, or no light barrier was passed after 4,000 ms. During the early stage of data acquisition, we noticed that the feedback for too-early responses was not working properly due to a programming error, which was fixed upon notice. However, in the final data set, we have 43 “too-early” trials (out of 19,200 trials) across five individuals where feedback did not work properly. Since the respective individuals did not show unusual data, we manually recoded these trials as scratch trials and proceeded with the data analyses. Excluding or including these five individuals did not change our conclusions. Error feedback was also provided when participants returned to the central touch-sensitive key before passing through a light barrier during a trial. Participants were instructed to perform quick gesture movements to prevent lifting their hand before making a decision (see also the trial exclusion procedure below).

#### Design and Analysis

Trials were classified as *RE* compatible/incompatible, *SE* compatible/incompatible, and *SR* compatible/incompatible (see Figure 2). Data were pre-processed in the following steps: Trials from the first block with either the U-shaped or inverted-U-shaped lever were discarded as practice. Afterward, we excluded scratch trials (6.38% overall): Those were trials with a response initiation faster than 100 ms, a time-out after 4,000 ms, or trials where individuals returned to the touch-sensitive key during a trial. Additionally, we also discarded trials where the duration between gesture initiation and the crossing of the light barrier was longer than 150 ms, to avoid including trials where participants might have first lifted their hand before making a decision.

As in Experiment 1, we analyzed RTs and PEs. For RT analyses, only trials with correct responses were considered (discarding 6.08% of the trials). RTs and PEs were analyzed as in Experiment 1.

### Results

#### RTs

Median RTs are visualized in Figure 9A. The corresponding ANOVA indicated longer RTs for RE incompatible (848 ms) relative to compatible (688 ms) trials, *F*(1, 38) = 166.42, *p* < .001, $\eta_{p}^{2}=.75$ . Further, RTs were longer for SE incompatible (793 ms) relative to compatible (744 ms) trials, *F*(1, 38) = 50.54, *p* < .001, $\eta_{p}^{2}=.57$ . We observed a significant main effect of SR compatibility, *F*(1, 38) = 5.75, *p* = .022, $\eta_{p}^{2}=.13$ , with surprisingly longer RTs for SR compatible (773 ms) relative to incompatible (763 ms) trials. None of the two-way interactions turned out statistically significant, RE × SE compatibility: *F*(1, 38) = 2.45, *p* = .126, $\eta_{p}^{2}=.06$ ; RE × SR compatibility: *F*(1, 38) = 0.04, *p* = .844, $\eta_{p}^{2}<.01$ ; and SE × SR compatibility: *F*(1, 38) = 1.52, *p* = .225, $\eta_{p}^{2}=.04$ . Importantly, we again observed a three-way interaction that was qualitatively similar as the one we observed in Experiment 3, *F*(1, 38) = 38.32, *p* < .001, $\eta_{p}^{2}=.50$ . For RE compatible trials, we observed the SR incompatible condition to be harmful for performance in SE compatible conditions (25 ms), but advantageous in SE incompatible conditions (−43 ms). The reverse was true for RE incompatible trials. Here, we observed the SR compatible condition to be harmful for performance in SE compatible conditions (−59 ms), but advantageous in SE incompatible conditions (37 ms). The separate ANOVAs for each condition of RE compatibility yielded both significant two-way interactions, SR × SE interaction for RE compatible trials: *F*(1, 38) = 69.96, *p* < .001, $\eta_{p}^{2}=.65$ ; SR × SE interaction for RE incompatible trials: *F*(1, 38) = 17.73, *p* < .001, $\eta_{p}^{2}=.32$ .

**Figure 9.**
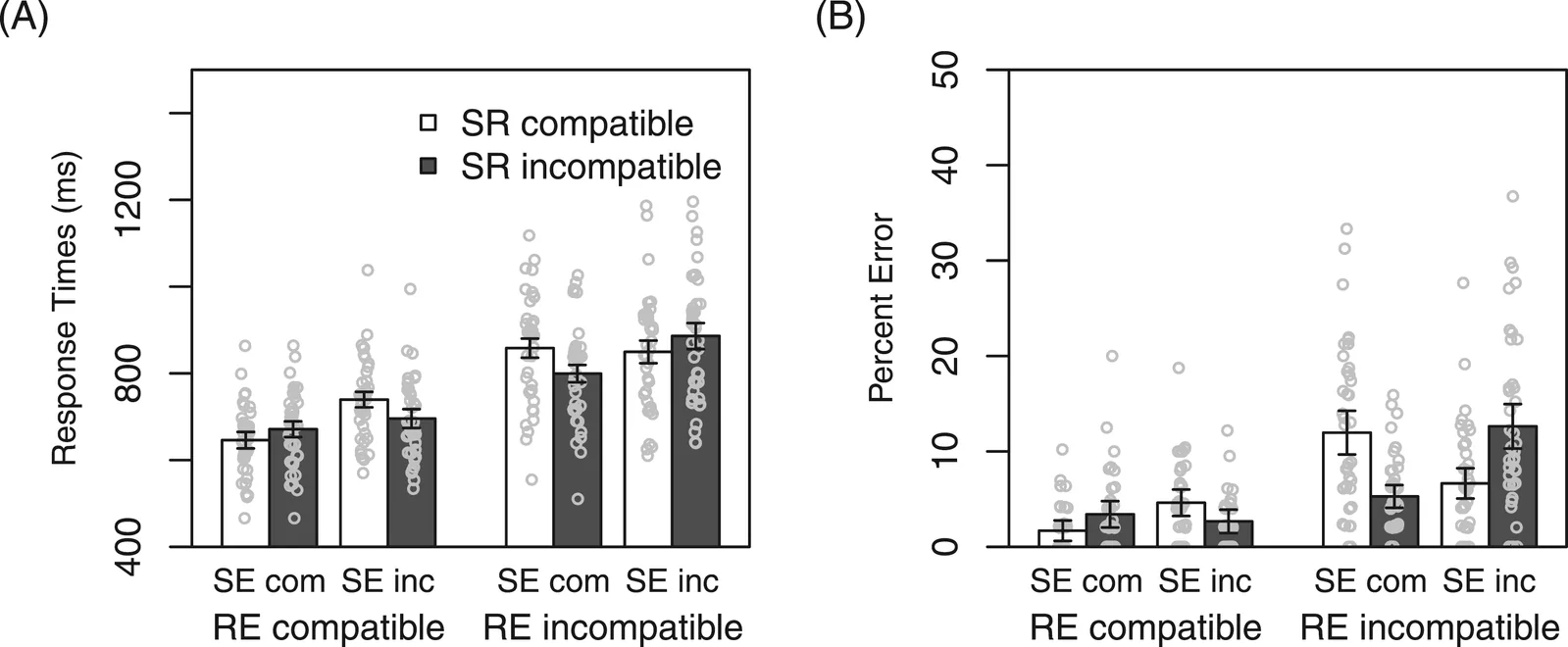
Median response times and percent error for Experiment 4. *Note.* Panel (A) shows the results for RTs as a function of RE, SR, and SE compatibility. Panel (B) shows the analogues results for PEs. White and dark gray bars represent compatible and incompatible SR relationships, respectively. Gray circles indicate individual observations. Error bars indicate 95% confidence intervals after removing between-participant variance (Morey, 2008)

#### PEs

Mean PEs are visualized in Figure 9B. The corresponding ANOVA indicated higher PEs for RE incompatible (9.1%) relative to compatible (3.1%) trials, *F*(1, 38) = 51.80, *p* < .001, $\eta_{p}^{2}=.58$ . PEs were higher for SE incompatible (6.6%) relative to compatible (5.6%) trials, *F*(1, 38) = 5.09, *p* = .030, $\eta_{p}^{2}=.12$ . The main effect of SR compatibility was not significant, *F*(1, 38) = 0.29, *p* = .590, $\eta_{p}^{2}=.01$ . The two-way interactions between SE and RE compatibility, *F*(1, 38) = 0.01, *p* = .928, $\eta_{p}^{2}<.01$ , and between SR and RE compatibility, *F*(1, 38) = 0.09, *p* = .769, $\eta_{p}^{2}<.01$ , did not turn out statistically significant. The interaction between SE and SR compatibility turned out statistically significant, *F*(1, 38) = 12.43, *p* = .001, $\eta_{p}^{2}=.25$ . Responses for SR compatible trials were more error prone relative to SR incompatible trials for the SE compatible condition (−2.5%). For SE incompatible conditions, a compatible SR relationship was advantageous (2.1%). Importantly, we observed a significant three-way interaction, *F*(1, 38) = 41.26, *p* < .001, $\eta_{p}^{2}=.52$ , with the qualitative pattern as observed for RTs. Separate ANOVAs for each condition of RE compatibility yielded significant interactions, for RE compatible trials: *F*(1, 38) = 12.70, *p* = .001, $\eta_{p}^{2}=.25$ ; for RE incompatible trials: *F*(1, 38) = 29.52, *p* < .001, $\eta_{p}^{2}<.44$ .

### Discussion

The results of Experiment 4 replicate those of Experiment 3. This contradicts some of our expectations, in particular we did not expect an RE compatibility effect with touchless gestures (see Janczyk, 2023; Janczyk et al., 2019). However, this effect was clearly observed with the present experiment setup. In addition, we also again observed a modulation of the SE × SR interaction by RE compatibility, similar to Experiment 3, that is, with opposite directions in RE compatible and in RE incompatible conditions.

## General Discussion

We investigated effects of SR, SE, and RE compatibility and their interactions when using a one-pivot lever across a series of four experiments. Many published studies using simple levers allowed to manipulate only two of the three compatibility relations independently. A study by Müsseler and Skottke (2011) overcame this limitation by using two different levers. As is visualized in Figure 2, this allowed the authors to manipulate all three relations independently. With this setup, differences for the SR × SE interaction occurred depending on RE compatibility. In RE compatible conditions, SR compatibility facilitated performance when the SE relation was compatible as well. If the SE relation was incompatible, however, the effect of SR compatibility was reversed. In stark contrast, SR and SE compatibility had no effect in RE incompatible conditions. Because this observation was based on a small sample of *n* = 10 participants in the original study, Experiment 1 aimed to replicate it with a larger sample. A further conceptual replication was presented as Experiment 2, and Experiments 3 and 4 are variations where we tried to increase (Exp. 3) and eliminate (Exp. 4) the effect of RE compatibility.

### Summary of Main Results

The results of Experiments 1 and 2 are straightforward: The just summarized pattern reported by Müsseler and Skottke (2011) was replicated with a larger sample size with respect to RTs and PEs, independent of whether the effect movement was discrete (Exp. 1) or continuous (Exp. 2). Note that in an additional set of analyses, we also performed Bayesian ANOVAs to test for an absence of the SR × SE interaction in RE incompatible trials. The corresponding Bayes Factors *BF*_10_ were calculated in JASP 0.19.1 (JASP Team, 2024), with the full ANOVA model tested against the ANOVA model without an interaction. For RTs in both Experiments 1 and 2, we observed evidence in favor of the null hypothesis; *BF*_10_ = 0.25 and *BF*_10_ = 0.23, respectively. For PEs, we observed evidence in favor of the null hypothesis in Experiment 1, but a slight tendency for the alternative in Experiment 2; *BF*_10_ = 0.23 and *BF*_10_ = 1.7, respectively. We conducted these analyses to comply with our pre-registration. In the main text, however, we based our analyses and conclusion on the frequentist framework, matching with the original study by Müsseler and Skottke (2011), while avoiding inferential inconsistencies by selectively mixing Bayesian and frequentist inference (Dienes, 2024; Schreiner & Kunde, 2024).

By itself, this replication is an important result showing that the original report was not a result of chance. Interestingly though, we observed an interaction of SR and SE compatibility in RE incompatible conditions with respect to PIEs and PChs, suggesting that the result observed in Müsseler and Skottke (2011) may be specific to RTs and PEs.

The results differed markedly, however, in Experiment 3, when a continuous slider movement directly controlled the continuous lever movement. While the result was again the same for the RE compatible condition, the reversed pattern was now observed in the RE incompatible condition: SR compatible trials facilitated performance in SE incompatible trials, and they hindered performance in SE compatible trials. Experiment 4 used a touchless gesture response combined with the discrete effect movement (as in Exp. 1). The overall pattern of results was as in Experiment 3. This is notable, as we initially considered the touchless gesture movement to eliminate the RE compatibility effect (as was observed previously by Janczyk, 2023; Janczyk et al., 2019). However, the effect of RE compatibility was clearly present as well.

Please note that we discuss the present results, and in particular the three-way interaction, from the point of view that RE (in)compatibility is the moderating factor of the SR × SE interaction. This reasoning is motivated against the background of the ideomotor principle and since RE compatibility was the sole block-wise manipulation. From a statistical point of view, however, considering SR compatibility as moderating the interaction of SE and RE compatibility or SE compatibility as moderating the interaction of SR and RE compatibility is equally possible, of course.

### Interpretation of the Results and Their Implications

A first, and rather clear, observation is that of an RE compatibility effect when using a lever. This result has also been reported earlier (e.g., Janczyk, Pfister, & Kunde, 2012; Kloss & Kunde, 2026; Kunde et al., 2007, 2012; Müsseler et al., 2008; Wirth et al., 2016) and seems a rather stable feature, regardless of the specific lever type and response type (discrete, continuous, gestural). This reinforces the notion that this kind of tool-based sensorimotor perturbation (Hewitson et al., 2025) indeed can negatively impact performance in applied settings such as in laparoscopic surgery.

Second, RE compatibility effects with touchless gestures seem possible as they were observed in Experiments 4. This was not expected and it contrasts with previous reports of no or only small RE compatibility effects with touchless gestures (Janczyk, 2023; Janczyk et al., 2019). Frankly, we can only speculate about the reasons at present, as not many empirical studies of that kind are available thus far. One clear difference between the earlier studies and the current Experiment 4 is that the former used vertical gestures and effect movements, while the latter did so in the horizontal dimension. While, theoretically, it is possible that touchless gestures only yield RE compatibility effects in the horizontal dimension, it should also be noted that RE compatibility effects have been observed in the vertical dimension as well (Keller et al., 2010). Another difference is that effects were task-relevant in Experiment 4, while they were task-irrelevant in Janczyk (2023). Particularly, in all of the present experiments, the movement of the tip (i.e., the effect) determined if a current trial was correct or not. Yet, at the same time, the effects were also relevant in Janczyk et al. (2019), but still only a negligible influence of RE compatibility was observed with touchless gestures. Hence, task-relevance might be helpful, but cannot explain the large effects observed in the present Experiment 4. Future research directly comparing horizontal versus vertical dimensions and task-relevant versus task-irrelevant effect (and gesture) movements is required to carefully scrutinize the boundary conditions.

More complicated to interpret is the interaction between SR and SE compatibility depending on RE compatibility. As mentioned above, this observation of Müsseler and Skottke (2011) was the starting point for our series of experiments. On the basis of all four experiments, we tentatively suggest that the original observation, replicated in our Experiments 1 and 2, is only half the story. Rather, RE incompatibility seems to actually reverse the SR × SE interaction observed in RE compatible conditions under some circumstances.

More precisely, the strength of the RE compatibility effect might be the crucial moderator. In the original study, and in our Experiments 1 and 2, the RE compatibility effect was of medium strength, and the SR × SE interaction was not fully reversed, but qualitatively absent. Yet, when responses were more salient and natural as in Experiments 3 and 4, the RE compatibility effect was stronger and the SR × SE interaction was reversed. In fact, effect sizes for the RE compatibility effect in RTs were $\eta_{p}^{2}=.44$ and $\eta_{p}^{2}=.13$ in Experiments 1 and 2, while they were larger with $\eta_{p}^{2}=.79$ and $\eta_{p}^{2}=.75$ in Experiment 3 and 4, respectively.

A possibly related observation was reported by Janczyk, Pfister, and Kunde (2012). That study used a simple lever (as illustrated in Figure 1) in two experiments with RE and SE compatibility manipulated orthogonally. In Experiment 1, participants were prevented from seeing their hand and the typical RE and SE compatibility effects were observed. Experiment 2, however, increased the salience of the hand movement by instructions, and the SE compatibility effect somewhat reversed in RE incompatible trials: SE compatible trials now yielded longer RTs than neutral/incompatible trials.

However, the particular reason why stronger RE compatibility effects reverse the SR × SE interaction is more difficult to determine and currently unknown. One conjecture is that RE compatibility interacts with how participants represent the SR rules for correct responding. In some sense, the reversal of the SR × SE interaction has similarity to the reversed Simon effect with an incompatible SR mapping (Hedge & Marsh, 1975; Wühr & Biebl, 2009). One explanation for this is that an incompatible SR relation encourages participants to reverse the task rules. For example, while using the rule “respond with the key that matches the stimulus” in SR compatible trials, participants might reverse the rule to “respond with the key that does not match the stimulus” in SR incompatible trials. Something similar might be happening here, except that task rules are recoded in RE incompatible relative to compatible conditions. In this case, task rules might be organized in a “hierarchical” manner so that the rules associated with the handling of the lever type (i.e., with the RE compatibility conditions) can reverse lower level rules associated with specific stimuli and responses in case RE incompatibility is sufficiently strong (i.e., in Experiments 3 and 4). This might be encouraged by consistently experiencing an incompatible RE relation, which was manipulated block-wise, and which might drive a general expectation that other compatibility relationships are inverted as well. However, these interpretations are post-hoc and must remain speculative at present. In contrast to the aforementioned studies on the reversal of the Simon task, compatibility mappings in the present study were not explicitly instructed as a rule, but resulted from a combination of the lever and stimulus type. Crucially, the general instruction “move the tip toward the + and away from the × ” was always the same throughout the experiment. Thus, it is certainly less obvious which task rules or compatibility relationships could have been recoded in the present case.

In essence, the present results highlight that it is not always the case that (spatially) compatible relations facilitate responses. Considering SR, SE, and RE compatibility alone, this holds true, of course, with some possible exceptions such as the lack of an RE compatibility effect with (vertical) touchless gestures (but see the discussion above). When multiple such relations are at play simultaneously, their effects and interactions on performance obviously become more complicated. This implies that intuitive and safe system design cannot be achieved by optimizing a single compatibility relation in isolation. Instead, system designers must jointly consider how users respond to stimuli, how movements are controlled, and how those movements affect the system. At present, we admittedly lack a clear explanation for *why* the incompatible RE condition sometimes overrides and sometimes reverses the SR × SE interaction. We suspect that an increase in the response’s salience is crucial, but more research is needed to provide a more process-oriented explanation.

### Limitations, Constraints on Generality, and Future Research

On a theoretical level, it is notable that we observed a strong RE compatibility effect in Experiment 4 with touchless gesture responses, while previous studies did not (Janczyk, 2023; Janczyk et al., 2019). At present, the reasons for this are unknown. Future research should aim to replicate this result in a simpler setting and identify the critical differences possibly moderating the presence and absence of RE compatibility effects with touchless gestures. This, however, is beyond the scope of the present manuscript.

Other limitations and constraints are more on a practically-relevant level. For example, the present experiments were conducted with untrained students. This choice was deliberate, as it allowed for a close replication and extension of the original study by Müsseler and Skottke (2011), which relied on a comparable participant sample. Given the simplicity of the task and the basic cognitive mechanisms under investigation, we believe that the present findings are not limited to the student population. However, we expect them to primarily generalize to individuals of similar age who have little or no prior experience with lever-based tools.

An important open question thus concerns whether the observed results generalize to trained populations. For example, surgeons performing laparoscopic procedures routinely operate RE incompatible levers and may therefore exhibit substantially reduced or even absent RE compatibility effects due to extensive training. One tentative possibility is that with sufficient training, RE compatibility becomes largely irrelevant, such that the interaction between SE and SR compatibility no longer depends, or only partially depends, on the RE relationship. If so, then it is possible that the interaction of SR and SE compatibility is identical for both RE compatible and RE incompatible trials, somewhat matching with the results for PIEs and PChs of Experiment 2 (see Figure 6).

Beyond participant characteristics, there are also constraints related to the experimental procedure. All experiments employed a virtual lever presented on a computer screen, and for reasons of experimental control, target stimuli were arranged horizontally along a single line (see Figure 4). This design choice required the lever to be rendered as a three-dimensional object that was slightly tilted away from the participant, resulting in a configuration that could not exist in the same form in a physical setup. A more realistic physical implementation would require positioning stimuli along a semi-circle with varying vertical heights. Thus, it remains an open question whether the present results fully generalize to physical levers and more naturalistic spatial arrangements.

## Key Points

• We replicate and extend Müsseler and Skottke (2011) to test how response–effect (RE), stimulus–response (SR), and stimulus–effect (SE) compatibility jointly influence lever tool performance.
• Across four experiments, we orthogonally manipulated RE, SR, and SE compatibility while varying input modality (button presses, continuous sliders, touchless gestures) and lever rotation type (discrete vs. continuous).
• Under RE compatible conditions, performance was improved when SR and SE compatibilities aligned. Under RE incompatible conditions, the SR × SE interaction disappeared with button press responses (Exp. 1 and 2) or reversed with continuous and gesture responses (Exp. 3 and 4).
